# Sphingolipidomic Analysis of *C. elegans* reveals Development- and Environment-dependent Metabolic Features

**DOI:** 10.7150/ijbs.30499

**Published:** 2019-11-08

**Authors:** Xiaoxiang Cheng, Xue Jiang, Kin Yip Tam, Gang Li, Jun Zheng, Hongjie Zhang

**Affiliations:** 1 Faculty of Health Sciences, University of Macau, Taipa, Macau SAR 999078, China; 2 Centre of Reproduction, Development and Ageing, University of Macau, Taipa, Macau SAR 999078, China

**Keywords:** *Caenorhabditis elegans*, sphingolipidomics, development, temperature, nutrition, LC/MS/MRM.

## Abstract

Sphingolipids (SLs) serve as structural and signaling molecules in regulating various cellular events and growth. Given that SLs contain various bioactive species possessing distinct roles, quantitative analysis of sphingolipidome is essential for elucidating their differential requirement during development. Herein we developed a comprehensive sphingolipidomic profiling approach using liquid chromatography-mass spectrometry coupled with multiple reaction monitoring mode (LC-MS-MRM). SL profiling of* C. elegans* revealed organism-specific, development-dependent and environment-driven metabolic features. We showed for the first time the presence of a series of sphingoid bases in *C. elegans* sphingolipid profiles, although only C17-sphingoid base is used for generating complex SLs. Moreover, we successfully resolved growth-, temperature- and nutrition-dependent SL profiles at both individual metabolite-level and network-level. Sphingolipidomic analysis uncovered significant SL composition changes throughout development, with SMs/GluCers ratios dramatically increasing from larva to adult stage whereas total sphingolipid levels exhibiting opposing trends. We also identified a temperature-dependent alteration in SMs/GluCers ratios, suggesting an organism-specific strategy for environmental adaptation. Finally, we found serine-biased GluCer increases between serine- versus alanine-supplemented worms. Our study builds a “reference” resource for future SL analysis in the worm, provides insights into natural variability and plasticity of eukaryotic multicellular sphingolipid composition and is highly valuable for investigating their functional significance.

## Introduction

SLs are involved in a wide array of cellular processes, such as protein modification, intracellular trafficking, proliferation, differentiation, apoptosis, angiogenesis, and stress response 1-3, in addition to their role as membrane components. Altered SL profiles are linked to many diseases including diabetes, cancer, neurodegenerative- and cardiovascular diseases as well as sphingolipidosis, a type of less common disease with altered SL metabolism 1-6. Yet, little is known about specific roles of SLs in the development of these diseases and their potential to serve as treatment targets. Furthermore, lack of knowledge about lipid system homeostasis remains a serious obstacle for analyzing their biological functions.

The SL metabolic pathway reveals a complex and often circular network of chemical reactions catalyzed by well conserved enzymes producing a multitude of diverse intermediates that interconnect 7, 8. With new technologies, such as mass spectrometry (MS), systematic studies on the relation of SL biosynthesis, metabolism and composition have become available in lower organisms 9, 10. Studies addressing the lipidome/sphingolipidome of various experimental systems ranging from tissue 11, 12 to cell 13-15 and further to organelles 16, 17 have generated some insight into cellular or subcellular functions of SLs. However, efforts addressing the interplay of SLs within the complete sphingolipidome in a whole animal level are still in their infancy 18, 19. To date, yeast* Saccharomyces cerevisiae* and* Drosophila* are the two organisms whose lipidomics has been studied at whole organism level, with tremendous advance in quantification of some of the SL classes (such as ceramide, hexosylceramide and phosphoethanolamine ceramide for fly), as well as in identification of the connections between cell behavior and SL alteration 12, 20-22.

Using *C. elegans* as a model system, significant progress has been made in functional and structural characterization of various SL species in the past decades. Specifically, ceramides are involved in IR-induced germ cell apoptosis 23 and starvation-induced stress response 24, and a subset of ceramides with C20-C22 FA chains plays an important role in the anoxia response 25. Lack of S1P signaling results in impaired neurotransmitter release 26, 27, whereas excess S1P signaling leads to developmental and reproductive defect 28. Glucosylceramides are required for establishment and maintenance of cellular polarity, which serves as a prerequisite of not only normal organ morphogenesis but also TOR-mediated postembryonic growth and development 7, 29. Although some subsets of sphingolipids were measured in these studies 7, 25, 30, sphingolipidome-wide quantification has not been carried out. More importantly, in contrast to a variety of sphingoid base backbones in yeast, *Drosophila* and mammalian, there is only one single sphingoid base in *C. elegans* with a unique C17 branched-chain fatty acid (FA) 31, resulting in a considerable decrease of the size of its sphingolipidome. Nonetheless, *C. elegans* contains all functionally distinct sphingoid derivatives, including simple SLs (e.g. sphinganine [Sa], sphingosine [So] and sphingosine 1-phosphate [So1P]); N-acyl-derivatives (e.g. ceramide [Cer] and ceramide 1-phosphate [C1P]); and complex SLs (e.g. sphingomyelin [SM] and glucosylceramide [GluCer]). This simplicity allows us to use *C. elegans* for the analysis of sphingolipid homeostasis under various conditions, a prerequisite for any functional analysis of specific SLs.

Here we conducted a systematic and comprehensive investigation on sphingolipidomics using* C. elegans* as a model organism. The regulation of SL composition during growth and development (with entirely different requirements for SL biosynthesis) 12, 22, at different temperatures (known to induce membrane lipid composition changes to maintain membrane fluidity) 32, 33, and under different nutritional conditions (subject to tight hormonal and signaling control) 34, 35 was investigated. We generated for the first time a comprehensive qualitative and quantitative map of the *C. elegans* SL profiles under these specific growth states. Importantly, this analysis provides a baseline characterization of the homeostatic biochemical network and establishes LC-MS-MRM based sphingolipidomics as a powerful platform for complementing genetics studies in worms.

## Materials and methods

### Chemical materials

The Ceramide/Sphingoid Internal Standard Mixture containing 25uM each of 10 compounds, including C17-sphinganine (C17-Sa), C17-sphingosine (C17-So), C17-sphingosine-1-phosphate (C17-So1P), C17-sphinganine-1-phosphate (C17-Sa1P), C12-ceramide (C12-Cer), C25-ceramide (C25-Cer), C12-ceramide-1-phosphate (C12-C1P), C12-SM, C12-glucosylceramide (GluCer), C12-lactosyl (β)-ceramide, was obtained from Avanti Polar Lipids (Alabaster, AL, USA). C18-1-deoxy-sphinganine (C18-DOSa), C18-1-deoxymethyl-sphinganine (C18-DOMSa) were also purchased from Avanti Polar Lipids, and dissolved as 25uM in DMSO and used as standard for detecting and quantifying deoxy-sphingoid bases. Other reagents used were as follows: methanol (LC-MS grade, Merck Millipore, MA, USA), acetonitrile (LC-MS grade, Fluka, USA), 2-propanol, ammonium acetate and potassium hydroxide (Sigma-Aldrich, St. Louis, MO, USA), formic acid (LC-MS grade, Fisher, USA), glacial acetic acid (Merck, Germany). Water was obtained from a Milli-Q Water Purification System from Millipore (Bedford, MA, USA).

### Worm culture and collection

Wild-type *C. elegans* (Bristol, N2) worms were cultured and maintained at 22°C on standard nematode growth media (NGM) seeded with OP50 bacteria unless indicated otherwise. Worm synchronization was carried out using a standard protocol as previously described 36. In brief, gravid hermaphrodites were collected by washing them off the NGM plates and reconstituted to total 3.5 ml using M9 buffer. The worm solution was mixed with a fresh mixture of 0.5 ml 10 N NaOH and 1 ml bleach to achieve a complete release of eggs from the worm body through a repeated shaking-and-vortexing step every 2 minutes for a total of 8-10 minutes. The eggs were washed by M9 for five times and then seeded onto NGM plates with proper density. The various stages were verified by visual inspection under a microscope. The L1-L4- and adult-stage worms were collected at respective time points of 9-10, 21-22, 31-32, 39-40 and 55-56 hours after egg treatment at 22°C. In addition, the worms grown at 16°C, 20°C and 22°C were collected at 71-72, 47-48 and 39-40 hours after egg treatment, respectively, for obtaining L4-stage worms. To collect the worms under different nutritional conditions, worms were cultured on NGM plates seeded with a mixture of OP50 with alanine or serine solution (final concentration is 15mg per plate), and harvested at L4 stage around 39-40 hours after egg treatment. The harvested worms were stored at -80°C until use.

### Sphingolipid extraction

The worm samples were thawed and homogenated firstly by a sonicator (Fisher Scientific, Model 505 sonic dismembrator, Fair Lawn, NJ, USA), and then freeze-dried (FreeZone 4.5 Freeze Dryer, Labconco, Kansas City, MO, USA) for 24 hrs. The extraction of SLs from *C. elegans* was carried out according to a modified Bligh and Dyer's method 37, 38. Briefly, 30 mg of worms were added with 2 ml of methanol (MeOH) and 1 ml of chloroform (CHCl_3_), vortexed for 1 min, and incubated in a water bath (TW12, Julabo, Allentown, PA) at 48°C for 24 hrs. The supernatant was collected and the solvent was evaporated by nitrogen blowing at room temperature. 75 μL of 1 M potassium hydroxide (in MeOH) was added into the residues and the mixture was sonicated for 5 min. After an incubation at 37°C for 2 hrs, 3 μL glacial acetic acid was added for neutralization followed by a phase separation generated by adding 1 mL CHCl_3_ and 2 mL water. Following a centrifugation at 2,500 rpm for 10 min, the upper layer (water layer) was carefully removed and discarded; and the chloroform layer was dried under vacuum and reconstituted with CHCl_3_-MeOH (2:1). The samples were centrifuged several times to further remove impurities before LC-MS analysis.

### LC-MS analysis

The SL separation and analyses were performed using a Waters Acquity ultra-high performance liquid chromatography (UPLC) system in tandem with Waters Xevo TQD triple quadrupole mass spectrometers (LC/MS/MS, Waters, Milford, MA, USA). The LC-MS system was equipped with a binary solvent manager and an autosampler. Waters Xevo TQ-MS was set in a positive electrospray mode for quantification of SLs coupled with multiple reaction monitoring (MRM). The capillary voltage and the source extractor voltage were set at 3.5 kV and 3 V, respectively, and the cone voltage was 40 V. The source and the desolvatation gas temperatures were maintained at 140°C and 500°C, respectively. The desolvatation and cone gas flows were 500 L/hr and 1 L/hr, respectively. Dwell time and inter-channel delay were set to 20 and 5 ms. Data acquisition, data analysis and instrument control were done by Empower Pro 6.0 (Waters, Milford, MA, USA), and MS system was controlled by Analyst 1.4.2 software (Applied Biosystems, MDSSciex, Foster City, CA, USA). The standards and samples were separated using the following two gradient elution chromatographic separations.

Method 1 (normal phase, NP) - A waters ACQUITY UPLC BEH Amide column (1.7 μm 2.1×100mm) with an in-line filter was maintained at 45°C. Mobile phase A consisted of CAN : CH_3_OH : CH_3_COOH = 97 : 2 : 1 (v/v) containing 5mM NH_4_OAC; mobile phase B consisted of CH_3_OH : CH_3_COOH = 99 : 1 containing 5mM NH_4_OAC. The gradient elution was as follows: 0 - 1 min, 100% A; 1 - 4 min, 100 - 0 % A; 4 - 8 min, 100 % B; 8 - 10 min, 100 - 0 % B; 10-12 min, 100% A. The flow rate was set at 0.4 ml/min.

Method 2 (reverse phase, RP) - A Waters ACQUITY UPLC BEH C18 column (1.7 μm 2.1×100mm) with an in-line filter was maintained at 40°C. Mobile phase A consisted of 0.1% formic acid in ACN/water (20:80, v/v); mobile phase B consisted of 0.1% formic acid in ACN/2-propanol (20:80, v/v). The gradient elution was as follows: 0-1 min, 70% A; 1-2.5 min, 60-30% A; 2.5-4 min, 30-20% A; 4-5 min, 20% A; 5-6.5 min, 20-10 % A; 6.5-6.6 min, 10-0 % A; 6.6-8 min, 100% B. The flow rate was set at 0.25 ml/min.

### Data processing and analysis

Data analysis including peak smoothing and integration of areas under the curves for each ions was performed by Masslynx software (Waters). The semi-quantitation of individual metabolites was normalized with the respective internal standards, and calculated with the formula as follows.

Analyte Conc.(μg/ml) in Worm Sample = Area (analyte MRM peak)×Standard Conc.(μg/ml) / Area (Standard MRM peak)

The level of statistical significance was set by one-way ANOVA assay (*p*<0.05) and T-test (*, *p*<0.05; **, *p*<0.01; ***, *p*<0.001; ****, *p*<0.0001). Multivariate and univariate analyses and partial least squares-discriminate analysis (PLS-DA) were used to identify features that differed markedly between different experimental groups, and performed based on the concentrations of SLs (>50 variables) by MetaboAnalyst 4.0 39. A feature was considered significant when it had a student's t-test p value < 0.05.

## Results

### Experimental design and optimization of LC-MS-MRM conditions for *C. elegans*

**Figure 1:**
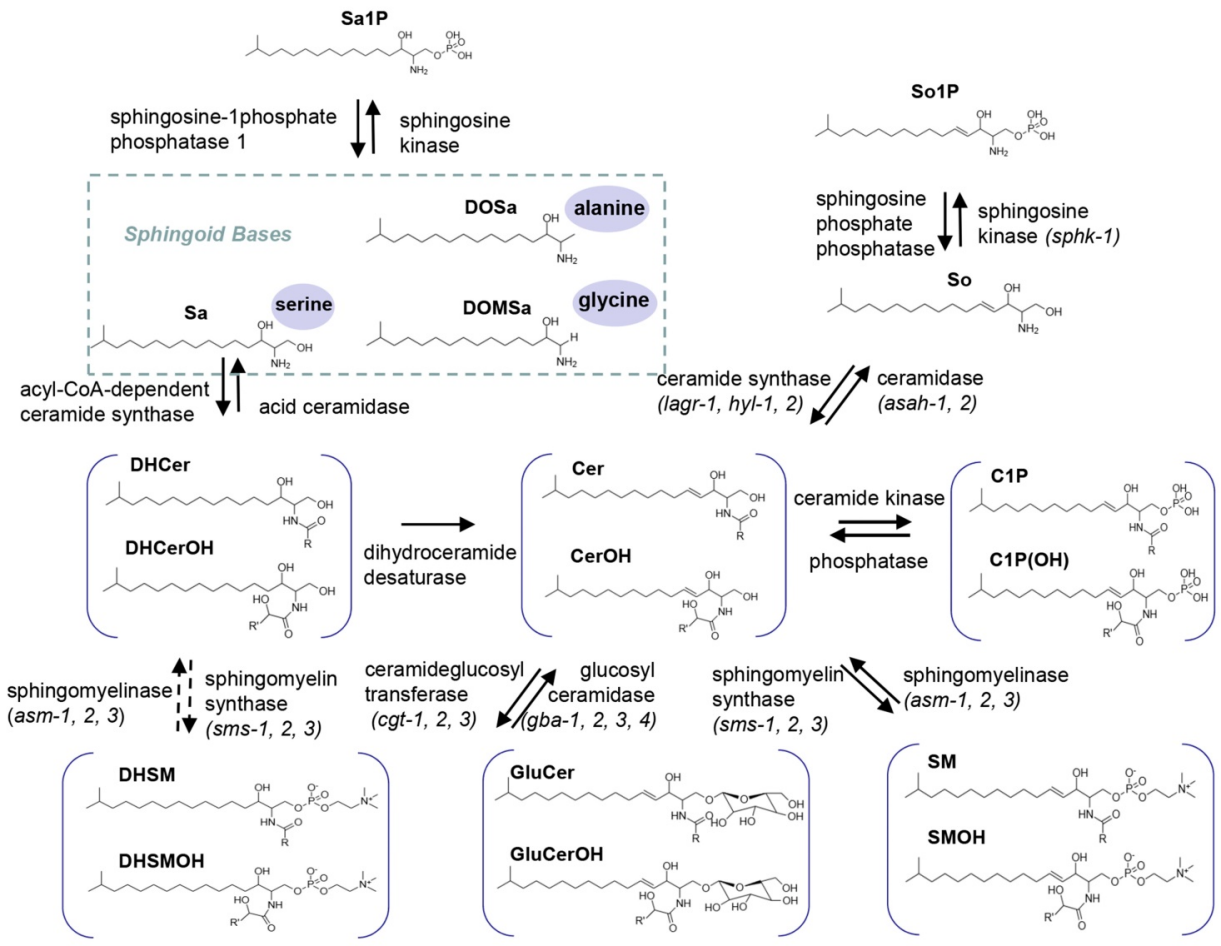
** Biosynthesis and turnover of three major categories of SLs in *C. elegans*. Top:** simple SLs; Sa, DoSa and DoMSa generated through condensation of a monomethyl branched-chain FA C15iso-CoA with serine, alanine and glycine, as well as So generated through Cer breakdown shown. Sa and So can be reversibly converted into Sa1P and So1P. **Middle:** N-acyl derivatives; in *C. elegans*, Sa will be mainly used for synthesis DHCer, which can be reduced to form Cer. Cer can be reversibly phosphorylated into C1P. **Bottom:** complex SLs. Cer can be reversibly synthesized into complex SLs such as GluCer and SM respectively. Molecular structure of each SL class with hydroxylated and non-hydroxylated derivatives and catalytic enzymes are shown. R indicate the side chain FA varying from C12 to C26.

Based on experimental protocols commonly applied in SL research 37, the effect of diverse growth and environmental factors on the SL composition of the *C. elegans* wild-type strain N2 was investigated by LC-MS-MRM. The tested factors include different growth phases (L1, L2, L3, L4, and adult stages), temperatures (16°C, 20°C, 22°C) and nutritional conditions (the presence or absence of amino acid alanine and serine). Targeted SL species include sphingoid bases, N-acyl-derivatives and complex SLs according to the SL metabolic pathway (Fig.1) 38. Of note, an abbreviated nomenclature for sphingoid bases and all other complex SLs is used throughout this work. The numbers of hydroxyls are designated by m (for mono-) and d (for di-) followed by the number of carbons, and the second number indicates the double bond. The numbers for carbons and double bonds for the second FA on structurally more complex SLs are indicated after a slash. For example, d18:0 stands for sphinganine, d18:1 stands for sphingosine and Cer (d17:1/22:0) stands for ceramide with a d17:1 sphingoid base and a C22 FA. Given that *C. elegans* N-acyl derivatives and complex SLs only contain sphingoid base synthesized from one single monomethyl branched chain C17iso FA, a “C” followed by a number is used when simply referring to a side chain length without necessity to distinguish detailed structural features on the second, normally saturated, FA chain of Cers and complex SLs.

To develop a robust technical platform for *C. elegans*-specific Sphingolipidomic analysis, we first optimized LC/MS scanning parameters (LC column, mobile phase gradient, elution protocol for SL species separation, ionization and fragmentation pattern) for each particular SL subclass using internal standard to achieve the best sensitivity and consistency. Comparative analysis of the ionization efficiency and separation precision with normal phase (NP)- and reverse phase (RP)-LC showed that S1P detection was significantly enhanced with RP-LC (Fig. S1A, Fig. S2), we therefore analyzed S1P in positive ion mode with RP-LC column and all other SLs under NP-LC mode. Notably, decrease of collision energy (CE) led to significant reduction of the signal intensity, optimal CE were therefore determined for each individual SL species (Fig. S1B and data were not shown). We also systematically tested various factors that might affect each step of SL extraction, including minimum amount of worm samples required for reliable detection, fractionation of apolar and polar lipids, the amount of KOH for lipid digestion and the solvent to reconstitute the SLs (Fig. S1C and data were not shown). To ensure the best range recovery of SLs and quality data acquisition, all SL extractions were carried out using one standard protocol with at least 30 mg of samples, without fractionation of apolar and polar lipids, 75 ul of KOH digestion and reconstitution in a mixture of 2:1 choloroform: methanol before analysis (see Methods for details).

### Molecular profiling of sphingolipids in *C. elegans*

**Figure 2:**
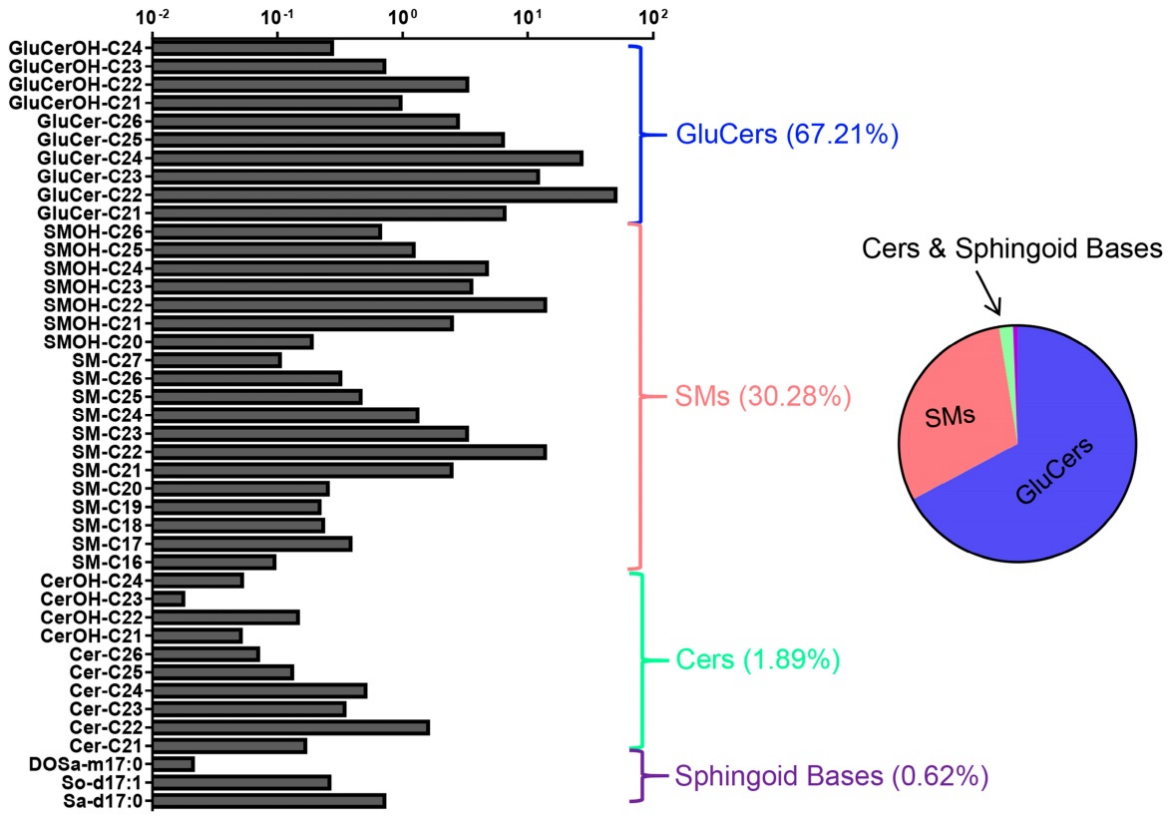
** Molecular composition of the wild-type *C. elegans* SL profile.** 42 quantified SL species in L1-stage N2 worms shown. The abundance of lipid species is depicted in milligram per microliter of worm samples on a logarithmic scale. Error bars indicate ± SDs, n=9 (3 biological replicates X 3 technical replicates). See Table S1 for detailed mass/charge (m/z) values for each individual species. Pie chart shows the relative concentrations cross different SL classes.

By LC-MS-MRM-based sphingolipidomic analysis we monitored about 180 molecular SL species in wild-type N2 animals, among which over 60 showed significant presence (Table S1). SL profiling shown in Fig. 2 depicted relative concentration of each individual species with higher abundance for L1-stage animals. We determined the absolute quantities of SL species by comparing the abundances of their precursors to the abundances of peaks of internal standard, and to make SL profiles comparable, we normalized the content of each individual SL species to the total dry weight of samples. We then calculated the total abundance of each SL class by summing up the abundances of individual species and calculated the percentile of each individual SL class to the total content of all measured SLs. Compositional analysis of total SL showed that L1-stage *C. elegans* contained 67.21% GluCers, 30.28% SMs, 1.89% Cers, and 0.62% free sphingoid bases (Fig. 2). Detection of each SL class is described in detail in following sessions.

**Figure 3:**
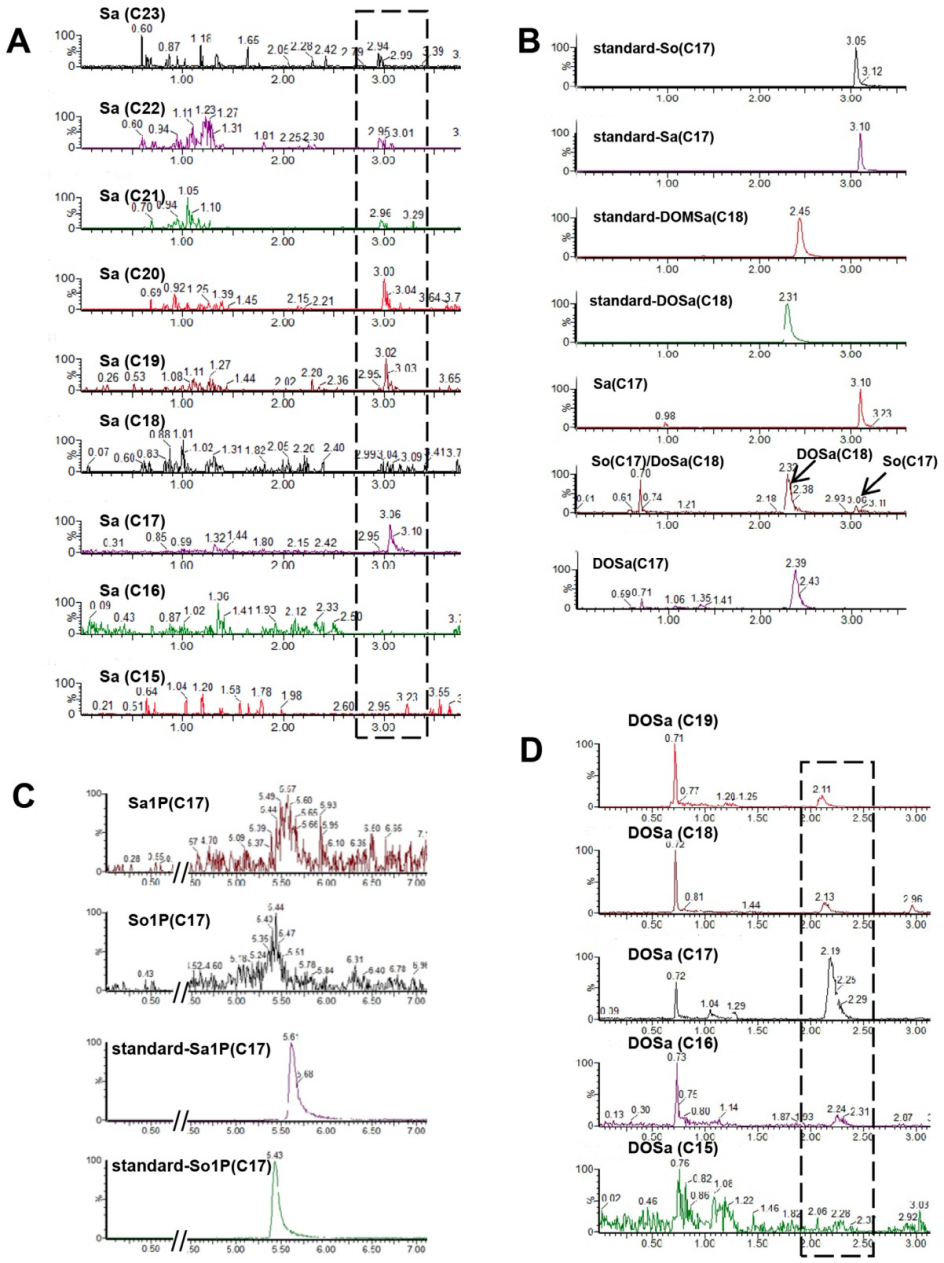
** The diversity of simple SLs in *C. elegans*.** (**A**) Representative spectra of Sa with various chain lengths. **(B)** Detection of Sa and So: by comparing with the elution peaks of internal standard, the profilings for worm samples were determined as Sa, DOSa (C18) and So (C17), and DoSa (C17). Note: So (C17) and DOSa (C18) shared the same channel (286.4<268.2) with distinct retention times, and Dosa (C17) could share the same ion channel and similar retention time with DoMSa (C18), however, it is very unlikely to be DoMSa (C18) due to the abscense of DoMSa (C17). **(C)** Detection of Sa1P and So1P by reverse phase LC. **(D)** Representative spectra of DoSa with various chain lengths. Note: the small shifts of elution time among the SLs with different chain lengths for Sa and DoSa are mainly caused by different injection time and different molecular weight. See Table S1 for detailed mass/charge (m/z) values for each individual species.

As the building block of SLs, more than seventy different sphingoid base backbones that vary in alkyl chain length (from 14 to 22 carbon atoms), degree of saturation and position of the double bonds, presence of a hydroxyl group at position 4 and branching of the alkyl chain have been reported 40. We attempted to detect *C. elegans* Sa and So with chain length ranging from 15 to 23 carbons, and found that d17:0, d19:0, d20:0 Sa were constantly detectable and more abundant than d21:0, d22:0 and d23:0 Sa, whereas d15:0, d16:0 and d18:0 Sa were almost undetectable (Fig. 3A). Specifically, the most abundant sphingoid base is d17:0 Sa while d17:1 So is the only compound within sphingosine subclass that can be detected (Fig. 3A, 3B), consistent with the notion that *C. elegans* contains the complex SLs derived from one single sphingoid base with a unique C17 branched-chain FA and also in line with the concept that sphingosine is mainly generated through the breakdown of complex SLs 31. The 1-phosphate metabolites of sphingoid bases, So1P/Sa1P (d16-d20), were systematically investigated in RP-LC mode, and only d17-So1P/Sa1P could be detected with low abundance in *C. elegans* (Fig. 3C). Deoxysphingoid bases (DSBs) are produced in mammals, including humans, and have been linked with the sensory neuropathy 41 and diabetes 42. C17 isobranched 1-deoxysphinganine were detected in wild-type *C. elegans exacts*, and worms fed with this compound caused cytoskeleton organization defects in intestine 43. We therefore specifically examined the atypical DSBs. The formation of two atypical deoxy-sphingoid bases 1-deoxy-sphinganine (DoSa) and 1-deoxymethyl-sphinganine (DoMSa) respectively relies on the condensation of alanine and glycine instead of serine with acyl-CoA. Provided that worms use the same FA substrate, the m17:0 DoSa and m16:0 DoMSa are expected to be generated (Fig. 1). DSB standard m18:0 DoSa and m17:0 DoMSa were eluted earlier than Sa and So probably due to slightly altered polarity and molecular weight. Through monitoring the intensity and shift of elution peaks together with the exact mass using NP and RP columns, we confirmed the presence of m17:0 DoSa, yet absence of m16:0 DoMSa in *C. elegans* (Fig. 3B). Additionally, m16:0, m18:0 and m19:0 DoSa were also successfully detected in *C. elegans* although their concentrations were very low (Fig. 3D). Thus, free sphingoid bases in *C. elegans* display some extent of structural diversity, and wild-type SPT has substantial affinity toward different length of FA and amino acids and is able to generate a multitude of sphingoid bases.

As the N-acyl derivatives of sphingoid bases, DHCer and Cer consist of a sphinganine and sphingosine backbone, respectively, linked with a FA through an amide bond. In line with previous reports 7, 23, 29, 37, DHCers and Cers contain a d17:0 or d17:1 sphingoid base with an amide-linked FA moiety varying from 20-26 carbon atoms with or without hydroxylation and C22 being the most abundant (Fig. 2, S3A, Table S1), further supporting the ability of this SL profiling technique. In *C. elegans*, Cers are 5-10 times more abundant than free sphingoid bases, yet 50 times less than complex SLs, and within the N-acyl derivative class, the abundance of each subclass is in an order of Cer>CerOH>DHCer>DHCerOH. Of note, regardless of detection of various sphingoid bases, DHCers and Cers derived from sphingoid bases other than C17, such as Cers (d18:1), Cers (d19:1), DHCer(d18:0) and DHCer(d19:0), or from DSBs, such as Cers (m17:0) and Cers (m19:0), were not found in *C. elegans* (data not shown).

As one of the downstream products of Cers, GluCer is generated through a direct transfer of a glucose moiety from a sugar-nucleotide (e.g. UDP-glucose) to the ceramide unit. GluCers (d17:1/C21:0-26:0) broadly existed in *C. elegans,* and C22-GluCer showed the highest abundance (Fig. 2, S3B, Table S1). SM consists of a ceramide unit with a phosphorylcholine moiety and could easily lose the phosphorylcholine group generating a precursor ion of m/z 184, therefore SMs were mainly quantified through the ion of m/z 184, but also validated by the ion of m/z 250.3, the signature ion of *C. elegans* sphingoid base. SMs contained the second FA varying from C16:0 to C26:0 with a total SM level comparable with that of GluCers and C22-SM being the most abundant (Fig. 2, S3C, Table S1).

In summary, the sphingolipidomics platform we developed featured a broad dynamic quantification range covering 3-4 orders of magnitude and a detection limit in the low ng/ml range. We estimated that the quantitative analysis covered 90% of the *C. elegans* sphingolipidome by monitoring lipid species constituting the 9 major SL classes in *C. elegans*. We excluded from our further analysis certain low abundant SL class and species that, for technical reasons, could not be robustly quantified (e.g. Cers [d17:0], Cers derived from DoSa sphingoid base [m17:0] and complex SLs with less abundant FA chain). Considering the low diversity of elements in SLs (H, C, N, O and P) as well as the long chains built by C-C bonds, the possibilities of multiple analytes possessing the same molecular formulas but different structures or atom arrangement exist. It is worth to note that the monomethyl-branched chain feature of the sphingoid bases and the precise location of double bonds and hydroxylations cannot be derived from our analytical approach.

### Influence of different factors on the *C. elegans* SL composition - general observation

**Figure 4:**
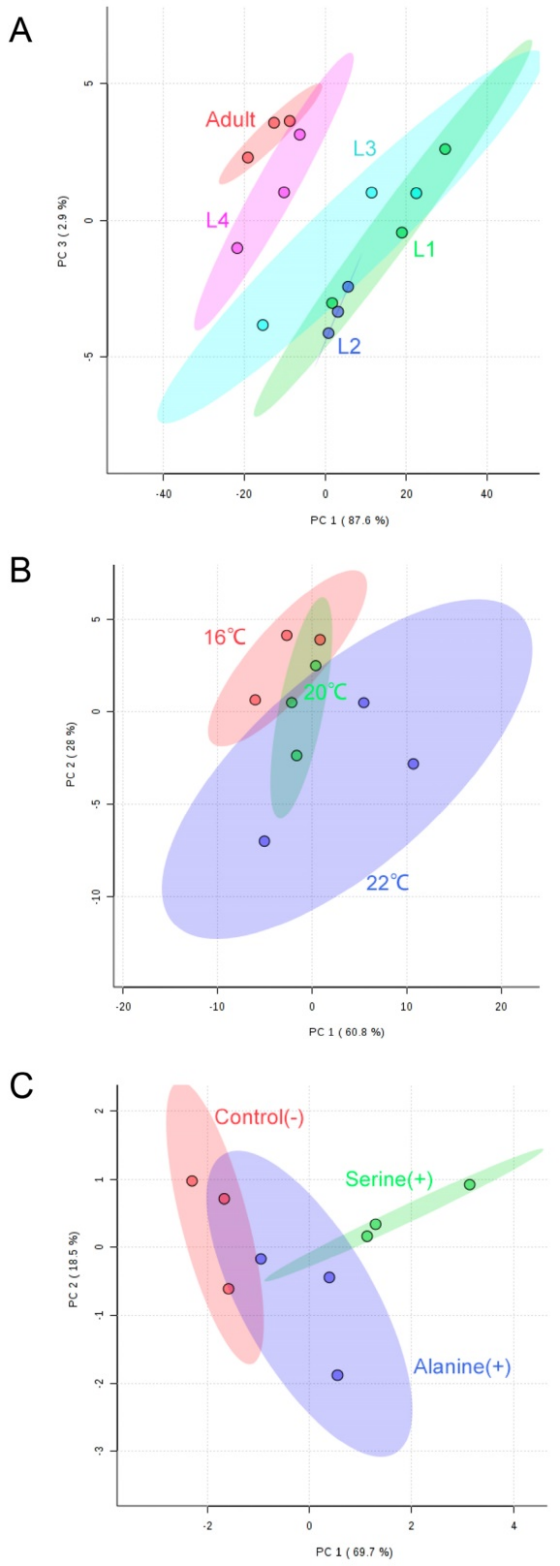
** Partial least square discriminant analysis (PLS-DA) of quantified SLs shows separation of N2 worms at different developmental and growth conditions.** (A) L1-L4 and adult-stage worms; (B) worms grown at 16°C, 20°C and 22°C; (C) worms fed with serine and alanine versus controls.

We next applied our targeted workflow to systematically measure SL composition variations during worm development, or when grown at different temperatures and under different nutritional conditions. To assess the main metabolic changes, we focused on a set of metabolic intermediates that show relatively high abundance and compared the normalized abundances of SL classes in worms under various conditions. Partial least square discriminant analysis (PLS-DA) of LC-MS/MS-based sphingolipidomics showed clear clustering of the metabolic profiles between different sample groups (Fig. 4). Multivariate analyses, as well as hierarchical clustering based on metabolite abundance revealed that the SL composition of the samples collected for different conditions exhibited a remarkable similarity yet with distinct metabolic features, thus confirming the reliability and the consistency of our approach.

### Developmental stage

**Figure 5:**
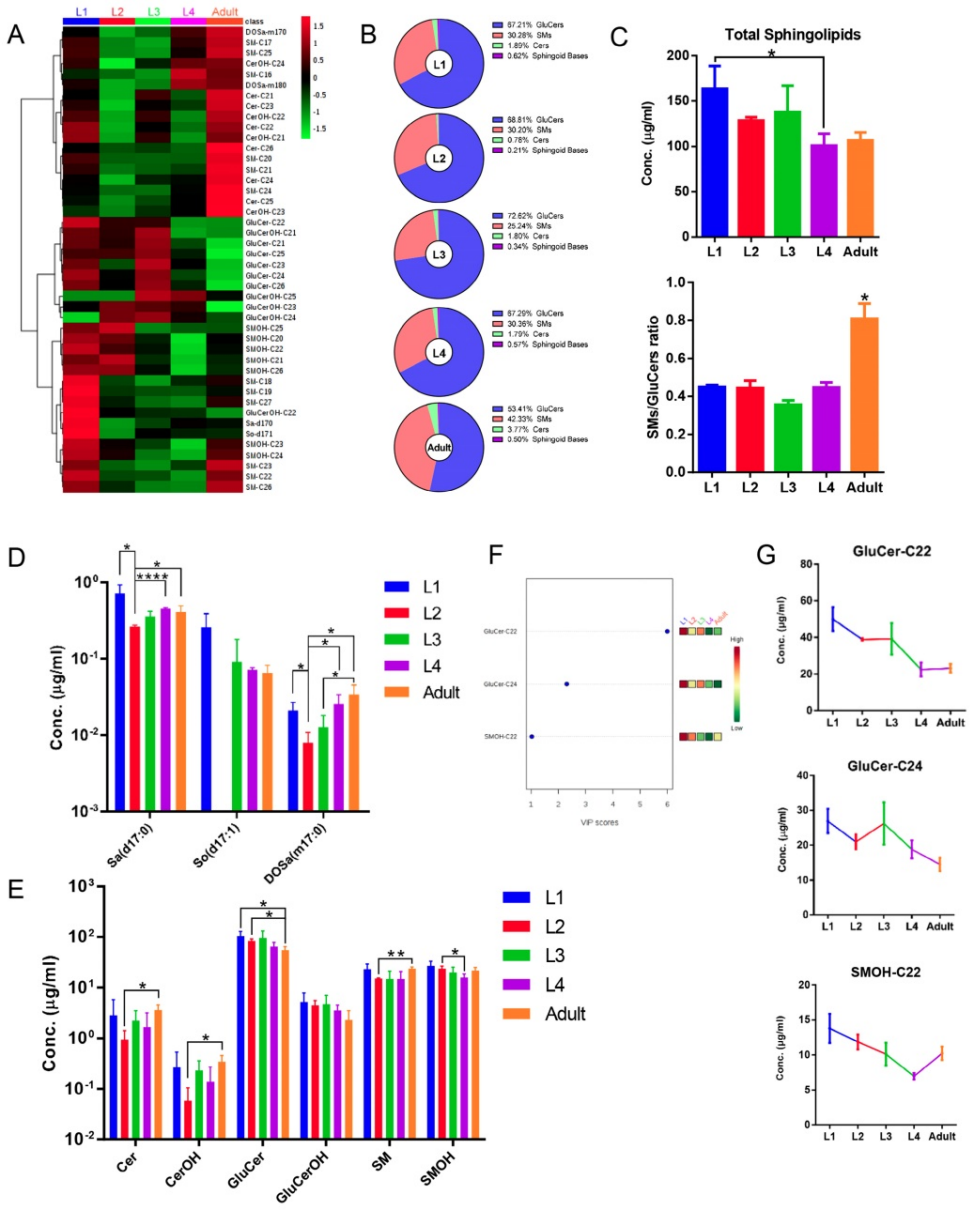
** Sphingolipidomic analysis reveals development-dependent metabolic features.** (A) Hierarchical heat map showing clustering of 44 SL species quantified in N2 worms at different stages. The color of each section is proportional to the significance of alteration of compounds. Green indicates lower and red indicates higher concentrations. Columns represent individual experiments and rows represent each quantified compound. (B) Stage-dependent changes of SL composition. (C) Stage-dependent variations of total SL level and SMs/GluCers ratio. (D) Quantification of the sphingoid bases of worms at different stages. (E) Quantification of the SL subclasses of worms at different stages. (F) VIP scores with expression heat map from PLS-DA analysis. Red and green indicate higher and lower levels, respectively. (G) Stage-dependent abundance changes of identified compounds with high VIP scores.

Hierarchical clustering based on metabolite abundance revealed striking differences in SL profile of worms at different developmental stages (Fig. 5A; Fig. S4). SL compositions changed remarkably throughout development, with SMs/GluCers ratios dramatically increasing from larval to adult stage, whereas total SL levels varied in an opposing trend (Fig. 5B, 5C). Intriguingly, each SL subclass fluctuated through development with different kinetics, which may be the main reason underlying the SL composition changes. Specifically, the abundance of sphingoid bases with 17 carbon for almost all five stages is roughly in an order of d17:0 Sa > d17:1 So > m17:0 DoSa, except completely devoid of d17:0 So at L2 stage (Fig. 5D). Sphingoid bases Sa and DoSa, together with majority of Cers, showed a similar variation trend: displaying the highest level in L1-stage animals and the lowest in L2-stage animals, and gradual increases comparing L2 with later stages (Fig. 5A, 5D, 5E). GluCers exhibited a mild continuous decrease trend from L1 to adult stage with the least at adult stage, while levels of SMs showed a gradual and slight reduction at larval stages followed by a mild increase from L4- to adult stage (Fig. 5A, 5E). Additionally, the variable importances in the projection (VIP) values were computed to identify SL species contributing to the observed separation of five worm stages of the PLS-DA model. Significantly, identified SLs with VIP value greater than one fell into several most abundant species (Fig. 5F), and they showed distinct stage-dependent curves yet with some similarity (Fig. 5G). One potential explanation for a growth-dependent differential metabolic kinetics for different SL classes could be that the cellular levels determine the amplitude of variation or the “susceptibility” of SL subclasses to growth. For example, SM and GluCer are present in concentrations that are 1-2 order of magnitude higher than those of Cer, therefore small changes in these compounds can result in profound changes in ceramide. Similarly, immediate hydrolysis of only 3-10% of newly generated Cer may double the levels of sphinganine.

**Figure 6:**
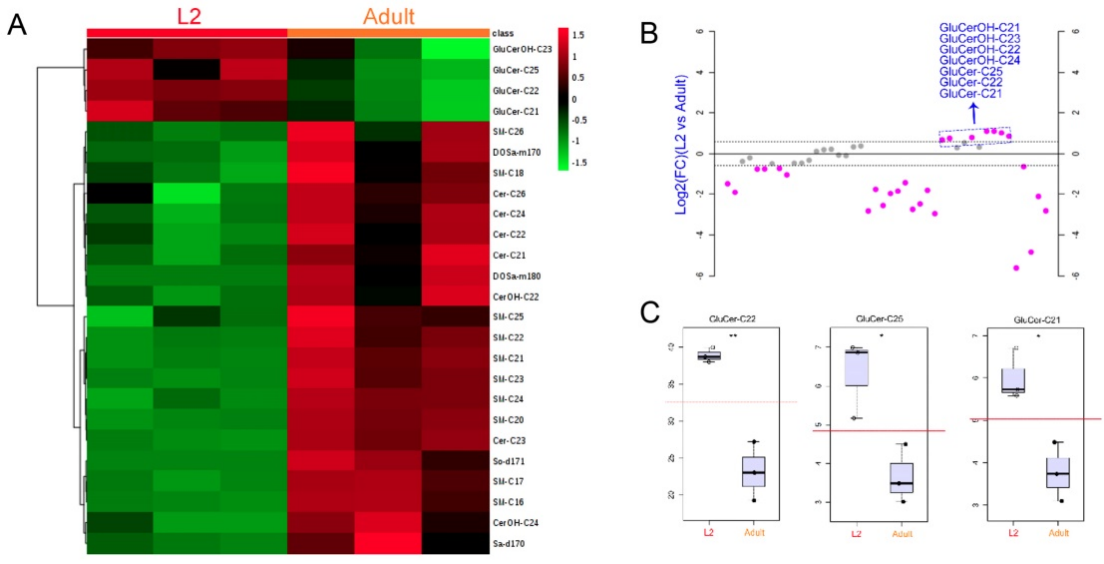
** Hierarchical cluster analysis of SLs in L2- and adult-stage worms.** (A) Clustered heat map of SLs showing a significant abundance increase for 21 out of 25 identified species and a decrease of 4 GluCer species comparing L2 and adult stages. (B) Ratio of quantified SLs in L2 versus adult N2 animals showing 7 GluCer compounds out of 44 SL species were more abundant in L2 than adult stage worms. (C) Dot plot of normalized abundance of three SL compounds in L2 and adult stage animals.

We next compared the SL compositions of L2- and adult-stage animals in more detail. Hierarchical clustering analysis of the two stages show a significant increase of majority of SL species from L2 to adult stage (Fig. 6A), whereas a striking decrease was observed in 7 GluCer species (Fig. 6B). This result was also reflected in stage-dependent SL composition changes (Fig. 5B). These data might indicate that, on one hand, GluCers are largely demanded during the early larval development, in line with the identified roles of GluCer in growth and epithelial polarity 7; on the other hand, the specific requirement of GluCer during L2 stage may attributable to the crosstalk between GluCer with sterol, such as dauer hormone 44.

### Temperature

**Figure 7:**
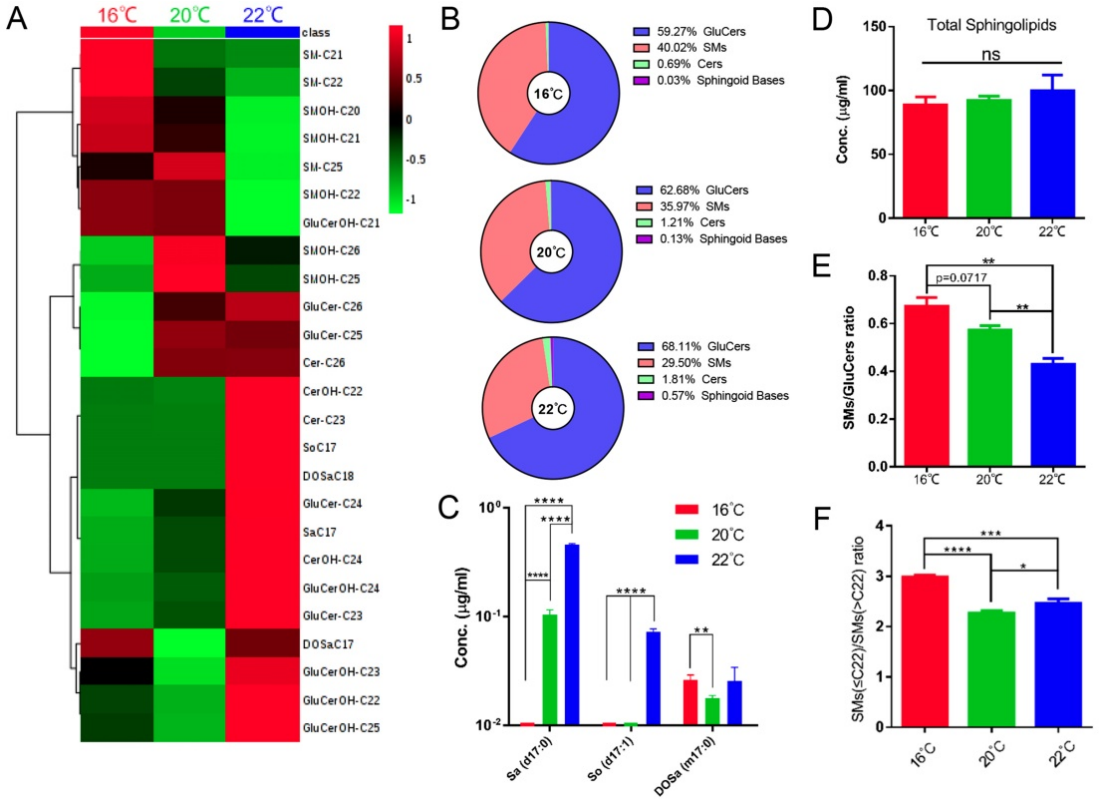
** Sphingolipidomic analysis reveals temperature-dependent metabolic features.** (A) Hierarchical heat map showing clustering of 25 SL species with significant variations at different temperatures (p<0.05). The color of each section is proportional to the significance of alteration of compounds. Green indicates lower and red indicates higher concentrations. Columns represent individual experiments and rows represent each quantified compound. (B) Temperature-dependent changes of SL composition. (C) Quantification of the sphingoid bases of worms grown at different temperatures. (D)(E)(F) Temperature-dependent variations of total SL level and SMs/GluCers and SMs(<22)/SMs(>22) ratios.

The effect of temperature on the SL composition was examined by growing* C. elegans* at 16°C, 20°C and 22°C. As shown in Fig. 7A, 7B and Fig. S5, SL compositions were strikingly influenced by culturing temperatures, and the temperature dependence of individual metabolites was not uniform across the SL profile. The levels of d17:0 Sa and d17:1 So dramatically decreased with lowering temperature. Interestingly, m17:0 DoSa (C17) is the only sphingoid base that could be detected at 16°C, whereas d17:1 So could barely be detected at both 16 and 20 °C (Fig. 7C).

Remarkably, different classes of structurally more complex SLs exhibit different responses upon temperature changes. Cers seemed to increase with temperature rising, although not in a statistically significant manner probably due to low abundance. Most of GluCers were significantly more abundant at 22°C with little abundance changes between 16-20°C whereas levels of some SMs were gradually increased as the temperature drops, and consequently the ratio of SMs versus GluCers was much higher at 16°C than that at 20°C and 22°C (Fig. 7A-B, D-E). We next examined SM subclass more closely, and found a mild alteration in FA compositions with the ratio of shorter FA- versus longer FA-containing SMs [SMs(≤C22)/SMs(>C22)] increasing as temperature decreasing despite an unaltered total SM level (Fig. 7F). Given that SL mainly contain saturated FAs, varying the ratio of different subclasses of SLs and FA chain length might be the supplementary mechanisms for the adjustment of membrane properties, in addition to the predominant mechanisms of changing desaturation in phospholipids.

### Nutrition

**Figure 8:**
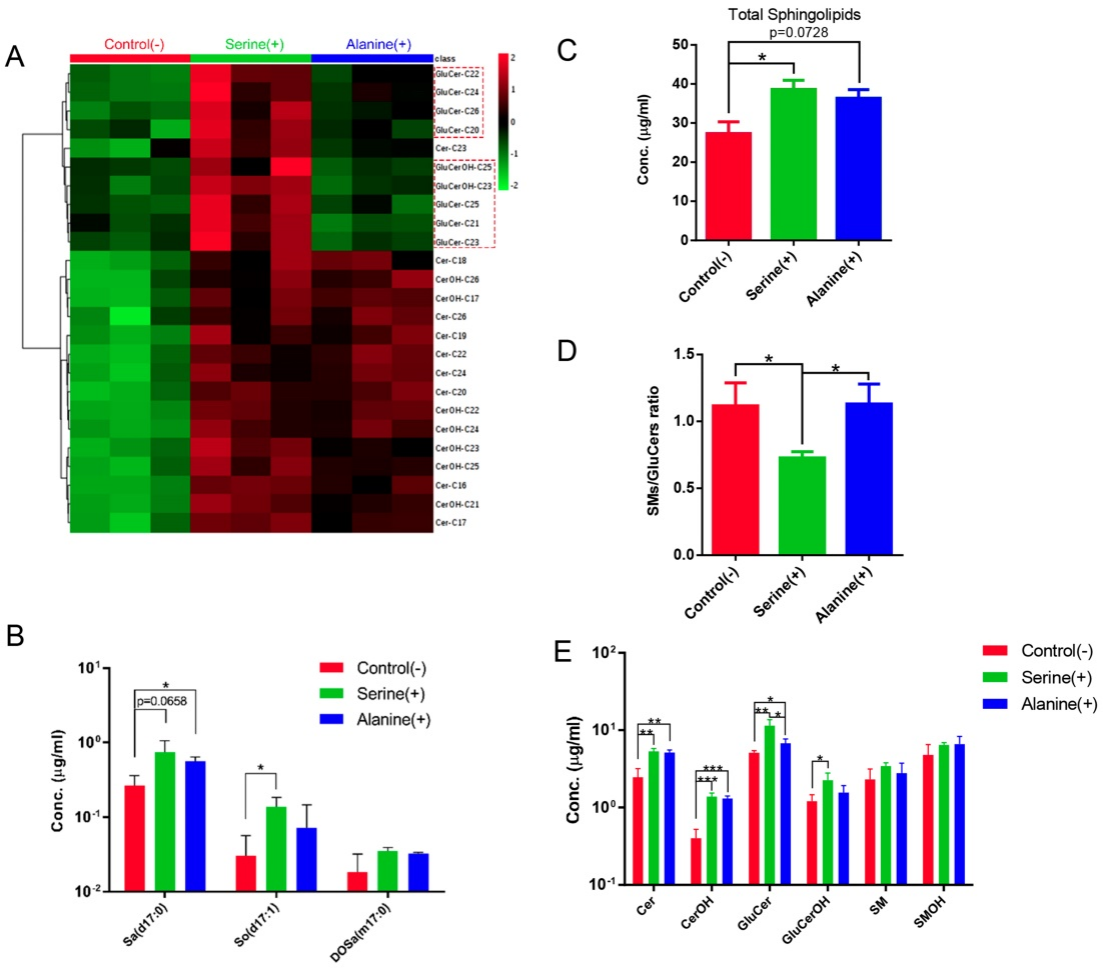
** Sphingolipidomic analysis reveals nutrition-dependent metabolic features.** (A) Hierarchical heat map showing clustering of 25 SL species with significant variations for control and serine- and alanine-supplemented groups (p<0.05). The color of each section is proportional to the significance of alteration of compounds. Green indicates lower and red indicates higher concentrations. Columns represent individual experiments and rows represent each quantified compound. (B) Quantification of the sphingoid bases of worms cultured under different nutritional conditions. (C-D) Nutrition-dependent variations on total SL level and SMs/GluCers ratio. (E) Quantification of the SL subclasses of worms cultured under different nutritional conditions.

We investigated the effect of amino acid supplementation on the SL composition of *C. elegans* by growing* C. elegans* on NGM plates supplemented with alanine or serine (see Materials and methods). Supplementation of alanine or serine led to the accumulation of majority of the detected SLs to a similar extent, including sphingoid bases and complex SLs (Fig. 8; Fig. S6). Interestingly, 9 GluCer species showed significant increase with serine-, but not much with alanine-, supplementation, therefore the ratio of GluCers/SMs was much higher upon serine supplementation, when compared to control and alanine-supplemented conditions (Fig. 8A, 8D). These data suggested that the enzymatic kinetics of different enzymes may respond differentially under different nutritional conditions. Specifically, the levels of d17:0 Sa and d17:1 So were elevated, however, not in a serine- or alanine- dependent manner respectively (Fig. 8B), suggesting that the overall increase of SL levels may not be caused by abundance change of the amino acid substrates rather by healthier “lifestyle” owing to the increased amino acids. Supplementation of free amino acids has been found to extend lifespan in yeast, worm, *Drosophila* and mouse 34, 45-48. Specifically, individual supplementation of 18 out of 20 amino acids (except phenylalanine and aspartate) extended lifespan and increased stress resistance *in C. elegans*, probably through activation of mitochondrial metabolism-mediated stress response pathway 45, which may in turn enhance SL production with or without upregulation of lipid metabolic pathways.

## Discussion

Investigating the intrinsic and extrinsic variability of SLs induced by different growth conditions in multicellular organisms is essential for better understanding biological roles and significance of SLs. This study established the LC-MS-MRM methodology as a valuable tool for molecular characterization of the *C. elegans* SL composition, and by this approach we quantitively and comprehensively described the abundance and composition of SLs under various growth and culturing conditions in *C. elegans*. The results revealed many development- and growth condition-dependent metabolic features of the *C. elegans* SL composition. Moreover, we confirmed the known characteristics of the *C. elegans* SL profile, e.g. the exclusive C17 sphingoid bases used to generate complex SLs, and identified novel characteristics, e.g. the detection of the DoSa bases in wild-type worms. In conclusion, this fundamental research of SLs in wild-type *C. elegans* under different developmental phases and growth conditions revealed key insights into the inherent flexibility of the *C. elegans* SL composition and might provide important references for further investigation on SL functions in *C. elegans*. However, several limitations of this resource should be noted, including: 1) we did not try to resolve the FA chain and monosaccharide configuration (e.g. straight vs. branched chain and glucose vs. galactose) and the related conclusions were drawn based on the previous studies; 2) some mass-to-charge (m/z) values remain conflicted with anticipated isobaric overlap (e.g. C18 DoMSa and C17 DoSa, even though the absence of C18DoMSa was speculated in *C. elegans*); 3) the amounts detected should not be considered as absolute concentration due to the structural difference between *C. elegans*-specific sphingolipid species and standards.

### Stage specific changes in SL composition

*C. elegans*, as a model organism for biological research in many fields including genetics, development and aging, has a well-defined developmental pattern. Once fertilized, *C. elegans* proceeds through early embryogenesis, four larval stages (L1-L4) - each separated by molting, and become a reproductive adult within 3-4 days. When unfavorable conditions (e.g. high temperature, stress, inadequate food, and over-crowding) persist, *C. elegans* undergoes growth arrest, either as an L1 larva or as a specialized L2 diapause form termed the dauer larva 49. Dauer larvae can survive for several months in this state and when conditions become favorable for growth, re-enter the life cycle at L4. Our experiments revealed mainly quantitative differences in SL compositions between different stages of animals, reflecting different requirement of SLs during worm growth. Specifically, the decreasing of many of SL levels between L1 and L2 larvae is a striking finding of this study. These changes could be partially attributable to the difference in total cell numbers between L1 and L2 stages. Taking the same weight amount from an L2 culture in comparison to an L1 culture means that the number of individuals that were used to start out with was different due to size and body mass differences. Comparing with bulk cell division and growth at L1 stage, very few cell divisions occur at L2 stage, although growth and cell divisions recover largely after L2, such as 143 cells grow from 2 precursors at L3 just for somatic gonad system. Therefore, the same weight of sample may contain more cells and bigger cell membrane surfaces at L1 than that in L2 stage. Moreover, these changes might also indicate L2-specific metabolic events. Although the worms used for lipid analysis were well fed, the dauer-inducing pheromones may reach the maximum at this stage which may affect gene expressions and lipid metabolism as well. Coincidently, genomic analysis of dauer entry as well as the dauer phase has revealed that the dauer state is associated with an increased expression of stress resistance genes, decreased metabolic rates, and altered lipid metabolism 50-52. In yeast, systematic characterization of proteomics and lipidomics at different growth phases has been carried out 22 and interesting stage dependent features on both the expression of SL biosynthetic enzymes as well as SL composition were identified.

### Effect of temperatures on SL composition

It is known that lipid compositions are highly dependent on temperature 53, which is confirmed in the present study. Growth temperature is the key factor for membrane fluidity and function, and different organisms use different strategies in response to temperature fluctuation. The bacterium *S. aureus* maintains membrane structure and fluidity through reducing the content of branched chain FAs when grown at a higher temperature 54, whereas a *Vibrio* strain regulates membrane fluidity by carrying out *cis* to *trans* isomerization of unsaturated FAs in response to temperature 55. In other bacteria, a combination of FA changes, including an increase in the level of unsaturation and a decrease in the average chain length of the fatty acyl chains occurs simultaneously as growth temperature changes 56. Among the strategies, modulating FA desaturation is one of the best-known effects of temperature stress, and has been observed in bacteria, yeast, plants, and *Drosophila* 56-58. Altering desaturation, for example in phospholipids, may also be use as the primary mechanism in response to temperature change by *C. elegans*. It would be interesting to monitor FA desaturation in phospholipids of worms grown at different temperatures. On the other hand, given that SLs mainly contain saturated FAs, changing SL composition and FA chain length of SLs may serve as the supplementary mechanism for worms to adapt to temperature changes.

Some lipid mixtures form membranes containing submicroscopic ordered lipid domains (rafts) that serve as platforms for signal transduction or protein sorting 59, 60. Lipid rafts are believed to be organized by SLs together with cholesterol and their sizes are highly temperature-dependent 61. *C. elegans* is thought to contain rafts 62, 63 however, its cell membranes contain little or no cholesterol, and its rafts may therefore consist exclusively of SLs 64. The ratio of complex SL, such as SMs/GluCers may be specifically important for altering the membrane physical properties, thereby governing microdomain formation, membrane budding and fusion, vesicle efflux, vesicular trafficking, and other aspects of membrane dynamics 60. Alternatively, shorter FA chain may exist in *C. elegans* in greater abundance to replace the structural functions ascribed to cholesterol in other animal cell membranes and they may take over the regulation of membrane fluidity and permeability as significant changes in the ratio of shorter SMs(FA≤C22) versus longer SMs(FA>C22) were detected responding to growth temperature 62-64.

### Effect of Dietary amino acids on SL composition

L-Serine is required to synthesize membrane lipids such as phosphatidylserine and SLs. Profound metabolic and physiological changes have been observed through manipulating serine content in various organisms. Deprivation of external L-serine through genetic depletion of L-serine biosynthetic enzyme leads to the generation of DSBs, including DoSa in mouse embryonic fibroblasts, which can be further suppressed or potentiated by supplementing exogeneous L-serine or increasing the ratio of L-serine to L-alanine, respectively 65. Furthermore, this serine-deprived condition significantly reduces levels of sphinganine, dihydroceramide, ceramide, and hexosylceramide while retaining levels of sphingomyelin, sphingosine, and sphingosine 1-phosphate 65. The accumulation of DSBs has been linked to Hereditary sensory and autonomic neuropathy type 1 41, 66, with the observation that high dose L-serine supplementation lowers plasma DSB levels and alleviates neuronal symptoms in HSAN1 patients, whereas alanine increases DSB levels and worsens peripheral neuropathy 67. Interestingly, increased serine synthesis also leads to the accumulation of SLs in aneuploid yeast cells, and impaired serine synthesis enhances sensitivity to inhibition of SL synthesis 68. Here, in this study, supplementation of L-serine or L-alanine did not affect the SL composition significantly, although total SL levels were increased. This result may not be surprising given that, under wild-type condition, there are probably enough substrates including both C17iso FAs and L-serine or L-alanine in the pool of free FAs and amino acids and it is the enzyme SPT that determines substrate specificity. Supplementation of free amino acids have been found to extend lifespan in yeast, worm, *Drosophila* and mouse 34, 45-48. Therefore, total SL level elevation observed here is compatible with the diet-mediated homeostatic regulation.

## Supplementary Material

Supplementary figures and tables.

## Abbreviations

CE: collision energy
Cer: ceramide
DOSa: 1-deoxy-sphinganine
DOMSa: 1-deoxymethyl-sphinganine
DSB: 1-deoxysphingoid base
FA: fatty acid
GluCer: glucosylceramide
LC: liquid chromatography
MRM: multiple reaction monitoring
MS: mass spectrometry
NGM: nematode growth media
NP: normal phase
PLS-DA: partial least squares-discriminate analysis
RP: reverse phase
Sa: sphinganine
So: sphingosine
SL: Sphingolipid
SM: sphingomyelin
Sa1P: sphinganine 1-phosphate
So1P: sphingosine 1-phosphate
VIP: variable importance in the projection

## References

[1] Breslow DK, Weissman JS (2010). Membranes in balance: mechanisms of sphingolipid homeostasis. Molecular cell.

[2] Blom T, Somerharju P, Ikonen E (2011). Synthesis and biosynthetic trafficking of membrane lipids. Cold Spring Harbor perspectives in biology.

[3] Bartke N, Hannun YA (2009). Bioactive sphingolipids: metabolism and function. Journal of lipid research.

[4] Hannun YA, Obeid LM (2017). Sphingolipids and their metabolism in physiology and disease. Nature reviews Molecular cell biology.

[5] Meikle PJ, Summers SA (2017). Sphingolipids and phospholipids in insulin resistance and related metabolic disorders. Nature Reviews Endocrinology.

[6] Olsen AS, Færgeman NJ (2017). Sphingolipids: membrane microdomains in brain development, function and neurological diseases. Open biology.

[7] Zhang H, Abraham N, Khan LA (2011). Apicobasal domain identities of expanding tubular membranes depend on glycosphingolipid biosynthesis. Nature cell biology.

[8] Futerman AH, Riezman H (2005). The ins and outs of sphingolipid synthesis. Trends in cell biology.

[9] Daum G, Lees ND, Bard M (1998). Biochemistry, cell biology and molecular biology of lipids of Saccharomyces cerevisiae. Yeast.

[10] Santos AX, Riezman H (2012). Yeast as a model system for studying lipid homeostasis and function. FEBS letters.

[11] Fan M, Sidhu R, Fujiwara H (2013). Identification of Niemann-Pick C1 (NPC1) disease biomarkers through sphingolipid profiling. Journal of lipid research.

[12] Carvalho M, Sampaio JL, Palm W (2012). Effects of diet and development on the Drosophila lipidome. Molecular systems biology.

[13] Sampaio JL, Gerl MJ, Klose C (2011). Membrane lipidome of an epithelial cell line. Proceedings of the National Academy of Sciences.

[14] Valsecchi M, Aureli M, Mauri L (2010). Sphingolipidomics of A2780 human ovarian carcinoma cells treated with synthetic retinoids. Journal of lipid research.

[15] Van Meer G (2005). Cellular lipidomics. The EMBO journal.

[16] Klose C, Surma MA, Simons K (2013). Organellar lipidomics—background and perspectives. Current opinion in cell biology.

[17] Llorente A, Skotland T, Sylvänne T (2013). Molecular lipidomics of exosomes released by PC-3 prostate cancer cells. Biochimica et Biophysica Acta (BBA)-Molecular and Cell Biology of Lipids.

[18] Wenk MR (2005). The emerging field of lipidomics. Nature reviews Drug discovery.

[19] Wenk MR (2010). Lipidomics: new tools and applications. Cell.

[20] Guan XL, Cestra G, Shui G (2013). Biochemical membrane lipidomics during Drosophila development. Developmental cell.

[21] Klose C, Surma MA, Gerl MJ (2012). Flexibility of a eukaryotic lipidome-insights from yeast lipidomics. PloS one.

[22] Ejsing CS, Sampaio JL, Surendranath V (2009). Global analysis of the yeast lipidome by quantitative shotgun mass spectrometry. Proceedings of the National Academy of Sciences.

[23] Deng X, Kolesnick RJBc (2015). Caenorhabditis elegans as a model to study sphingolipid signaling. Biological chemistry.

[24] Cui M, Wang Y, Cavaleri J (2017). Starvation-induced stress response is critically impacted by ceramide levels in Caenorhabditis elegans. Genetics.

[25] Menuz V, Howell KS, Gentina S (2009). Protection of C. elegans from anoxia by HYL-2 ceramide synthase. Science.

[26] Chan JP and Sieburth DJJoN (2012). Localized sphingolipid signaling at presynaptic terminals is regulated by calcium influx and promotes recruitment of priming factors. Journal of neuroscience.

[27] Chan JP, Hu Z, Sieburth DJG (2012). Recruitment of sphingosine kinase to presynaptic terminals by a conserved muscarinic signaling pathway promotes neurotransmitter release. Genes & development.

[28] Mendel J, Heinecke K, Fyrst H (2003). Sphingosine phosphate lyase expression is essential for normal development in Caenorhabditis elegans. Journal of biological chemistry.

[29] Zhu H, Shen H, Sewell AK (2013). A novel sphingolipid-TORC1 pathway critically promotes postembryonic development in Caenorhabditis elegans. Elife.

[30] Cutler RG, Thompson KW, Camandola S (2014). Sphingolipid metabolism regulates development and lifespan in Caenorhabditis elegans. Mechanisms of ageing and development.

[31] Chitwood DJ, Lusby WR, Thompson MJ (1995). The glycosylceramides of the nematode Caenorhabditis elegans contain an unusual, branched-chain sphingoid base. Lipids.

[32] Mendoza Dd (2014). Temperature sensing by membranes. Annual review of microbiology.

[33] Holthuis JC, Menon AK (2014). Lipid landscapes and pipelines in membrane homeostasis. Nature.

[34] Newgard CB (2012). Interplay between lipids and branched-chain amino acids in development of insulin resistance. Cell metabolism.

[35] Mirisola MG, Taormina G, Fabrizio P (2014). Serine-and threonine/valine-dependent activation of PDK and Tor orthologs converge on Sch9 to promote aging. PLoS genetics.

[36] Brenner SJG The genetics of Caenorhabditis elegans. 1974; 77: 71-94.

[37] Bligh EG, Dyer WJ (1959). A rapid method of total lipid extraction and purification. Canadian journal of biochemistry and physiology.

[38] Zhang H, Abraham N, Khan LA (2015). RNAi-based biosynthetic pathway screens to identify *in vivo* functions of non-nucleic acid-based metabolites such as lipids. Nature protocols.

[39] Chong J, Soufan O, Li C MetaboAnalyst 4.0: towards more transparent and integrative metabolomics analysis. 2018; 46: W486-94.

[40] Sang S, Kikuzaki H, Lapsley K (2002). Sphingolipid and other constituents from almond nuts (Prunus amygdalus Batsch). Journal of agricultural and food chemistry.

[41] Penno A, Reilly MM, Houlden H (2010). Hereditary sensory neuropathy type 1 is caused by the accumulation of two neurotoxic sphingolipids. Journal of biological chemistry.

[42] Bertea M, Rutti MF, Othman A (2010). Deoxysphingoid bases as plasma markers in diabetes mellitus. Lipids health dis.

[43] Hannich JT, Mellal D, Feng S (2017). Structure and conserved function of iso-branched sphingoid bases from the nematode Caenorhabditis elegans. Chemical science.

[44] Boland S, Schmidt U, Zagoriy V (2017). Phosphorylated glycosphingolipids essential for cholesterol mobilization in Caenorhabditis elegans. Nature chemical biology.

[45] Edwards C, Canfield J, Copes N (2015). Mechanisms of amino acid-mediated lifespan extension in Caenorhabditis elegans. BMC genetics.

[46] Alvers AL, Fishwick LK, Wood MS (2009). Autophagy and amino acid homeostasis are required for chronological longevity in Saccharomyces cerevisiae. Aging cell.

[47] Massie HR, Williams T (1985). Effect of sulfur-containing compounds on the life span of Drosophila. Age.

[48] D'Antona G, Ragni M, Cardile A (2010). Branched-chain amino acid supplementation promotes survival and supports cardiac and skeletal muscle mitochondrial biogenesis in middle-aged mice. Cell metabolism.

[49] Fielenbach N, Antebi A (2008). C. elegans dauer formation and the molecular basis of plasticity. Genes & development.

[50] Dorman JB, Albinder B, Shroyer T (1995). The age-1 and daf-2 genes function in a common pathway to control the lifespan of Caenorhabditis elegans. Genetics.

[51] Kenyon C, Chang J, Gensch E (1993). A C. elegans mutant that lives twice as long as wild type. Nature.

[52] Jeong PY, Kwon MS, Joo HJ (2009). Molecular time-course and the metabolic basis of entry into dauer in Caenorhabditis elegans. PLoS One.

[53] Spector AA, Yorek MAJJolr (1985). Membrane lipid composition and cellular function. Journal of lipid research.

[54] Wang LH, Wang MS, Zeng XA (2016). Temperature-mediated variations in cellular membrane fatty acid composition of Staphylococcus aureus in resistance to pulsed electric fields. Biochimica et Biophysica Acta (BBA)-Biomembranes.

[55] Okuyama H, Okajima N, Sasaki S (1991). The cis/trans isomerization of the double bond of a fatty acid as a strategy for adaptation to changes in ambient temperature in the psychrophilic bacterium, Vibrio sp. strain ABE-1. Biochimica et Biophysica Acta (BBA)-Lipids and Lipid Metabolism.

[56] Denich T, Beaudette L, Lee H (2003). Effect of selected environmental and physico-chemical factors on bacterial cytoplasmic membranes. Journal of microbiological methods.

[57] Li Q, Zheng Q, Shen W (2015). Understanding the biochemical basis of temperature-induced lipid pathway adjustments in plants. The plant cell.

[58] Overgaard J, Sørensen JG, Petersen SO (2005). Changes in membrane lipid composition following rapid cold hardening in Drosophila melanogaster. Journal of insect physiology.

[59] Lingwood D, Simons K (2010). Lipid rafts as a membrane-organizing principle. Science.

[60] Simons K, Gerl MJ (2010). Revitalizing membrane rafts: new tools and insights. Nature reviews Molecular cell biology.

[61] Pathak P, London E (2015). The effect of membrane lipid composition on the formation of lipid ultrananodomains. Biophysical journal.

[62] Sedensky MM, Siefker JM, Koh J (2004). A stomatin and a degenerin interact in lipid rafts of the nervous system of Caenorhabditis elegans. American Journal of Physiology-Cell Physiology.

[63] Rao W, Isaac RE, Keen JN (2011). An analysis of the Caenorhabditis elegans lipid raft proteome using geLC-MS/MS. Journal of proteomics.

[64] Kurzchalia TV, Ward S (2003). Why do worms need cholesterol?. Nature cell biology.

[65] Esaki K, Sayano T, Sonoda C (2015). L-Serine deficiency elicits intracellular accumulation of cytotoxic deoxy-sphingolipids and lipid body formation. Journal of biological chemistry.

[66] Eichler FS, Hornemann T, McCampbell A (2009). Overexpression of the wild-type SPT1 subunit lowers desoxysphingolipid levels and rescues the phenotype of HSAN1. Journal of neuroscience.

[67] Garofalo K, Penno A, Schmidt BP (2011). Oral L-serine supplementation reduces production of neurotoxic deoxysphingolipids in mice and humans with hereditary sensory autonomic neuropathy type 1. The Journal of clinical investigation.

[68] Hwang S, Gustafsson HT, Sullivan CO (2017). Serine-dependent sphingolipid synthesis is a metabolic liability of aneuploid cells. Cell reports.

