# Supplementary material for: Sphingolipidomic Analysis of C. elegans reveals Development- and Environment-dependent Metabolic Features
Source: Int J Biol Sci. 2019 Nov 8;15(13):2897–910. doi: 10.7150/ijbs.30499 (PMC6909964; doi:10.7150/ijbs.30499)
Supplement: Supplementary file 1 — Supplementary figures and tables. [file ijbsv15p2897s1.pdf]

Supplementary Materials

Sphingolipidomic Analysis of *C. elegans* Reveals Development- and Environment-dependent Metabolic Features

Xiaoxiang Cheng#, Xue Jiang#, Gang Li, Kin Yip Tam, Jun Zheng and Hongjie Zhang*

Figure S1

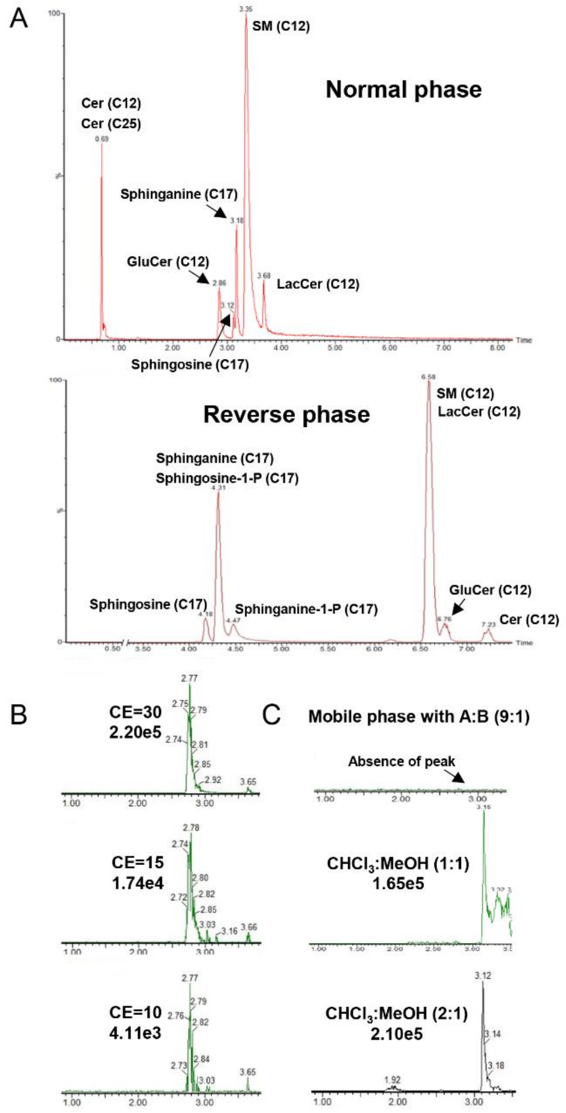

Figure S1: Optimization of LC-MS parameters for worm SL profile analysis. (**A**) LC-MS spectra of internal standard mixture obtained via NP and RP LC. RP-LC is significantly better for eluting Sa1P and So1P, but not for all other species. Therefore, RP-LC was used only for Sa1P and So1P detection and NP-LC is used for detection of all other SL species. (**B**) LC-MS spectra of internal standard GluCer-C12 under different collision energies. Collision energy of 30 generated the best ion signal. (**c**) LC-MS spectra of worm GluCer-C22 dissolved by different solvents. GluCer-C22 could not dissolve in mobile phase A:B (9:1), therefore no peak was detected at the presumed elution time. GluCer-C22 dissolved better in $CHCl_3$:MeOH 2:1 than in CHCl3:MeOH 1:1, therefore, higher intensity was detected when using $CHCl_3$:MeOH 2:1 as solvent.

Figure S2

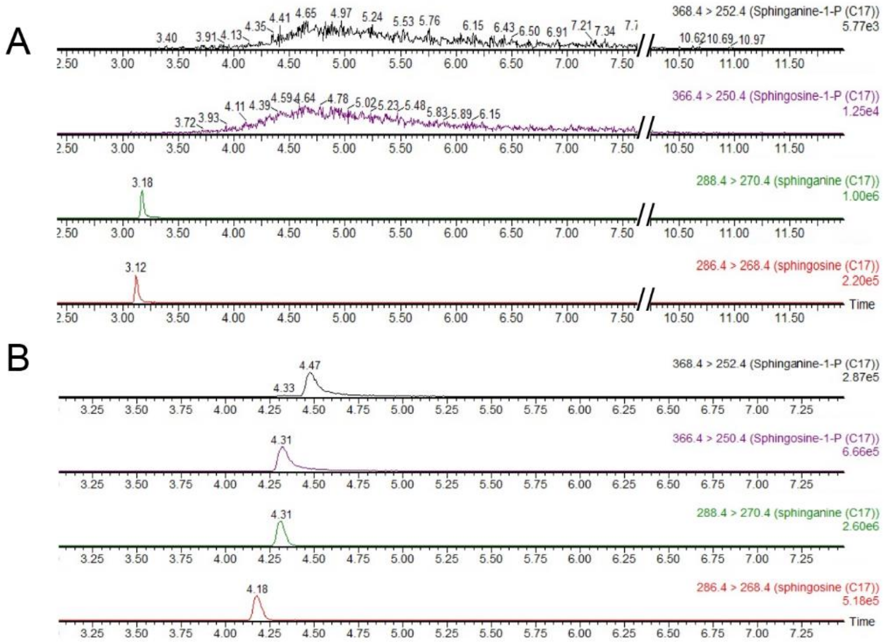

Figure S2: Detection of S1P using normal phase and reverse phase LC-MS. (**A**) LC-MS spectra showing the elution of d17:0 Sa, d17:1 So, Sa1P and So1P for standard using NP-LC. (**B**) LC-MS spectra showing the peak shifting of d17:0 Sa, d17:1 So, Sa1P and So1P using RP-LC column.

Figure S3

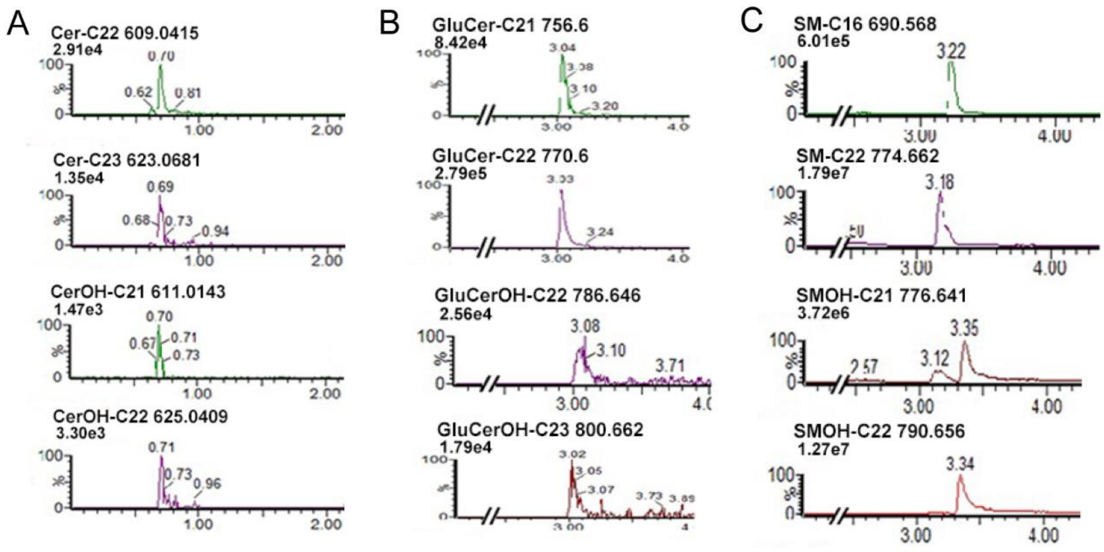

Figure S3: Representative SL LC-MS spectra of N2 worms. Spectra of (**A**) Cer/CerOH; **(B)** GluCer/GluCerOH; **(C)** SM/SMOH shown. SL species together with parent ions and intensities depicted. Note: the small shifts of elution time among different samples and standards are mainly caused by different injection time and different molecular weight.

Figure S4

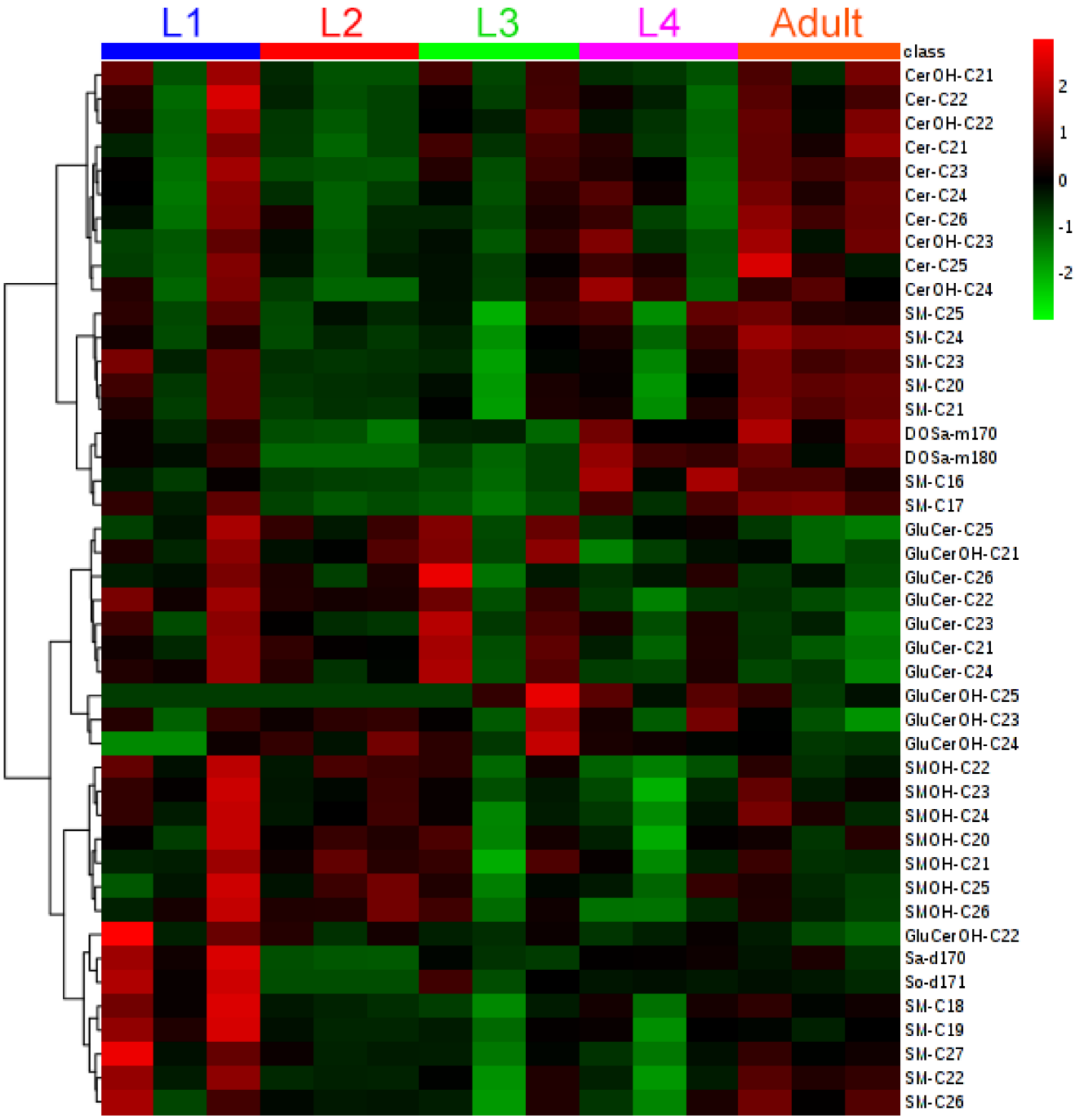

Figure S4: Hierarchical heat map showing clustering of 44 SL species quantified in N2 worms at different temperatures. The color of each section is proportional to the significance of alteration of compounds. Green indicates lower and red indicates higher concentrations. Columns represent individual experiments and rows represent each quantified compound.

Figure S5

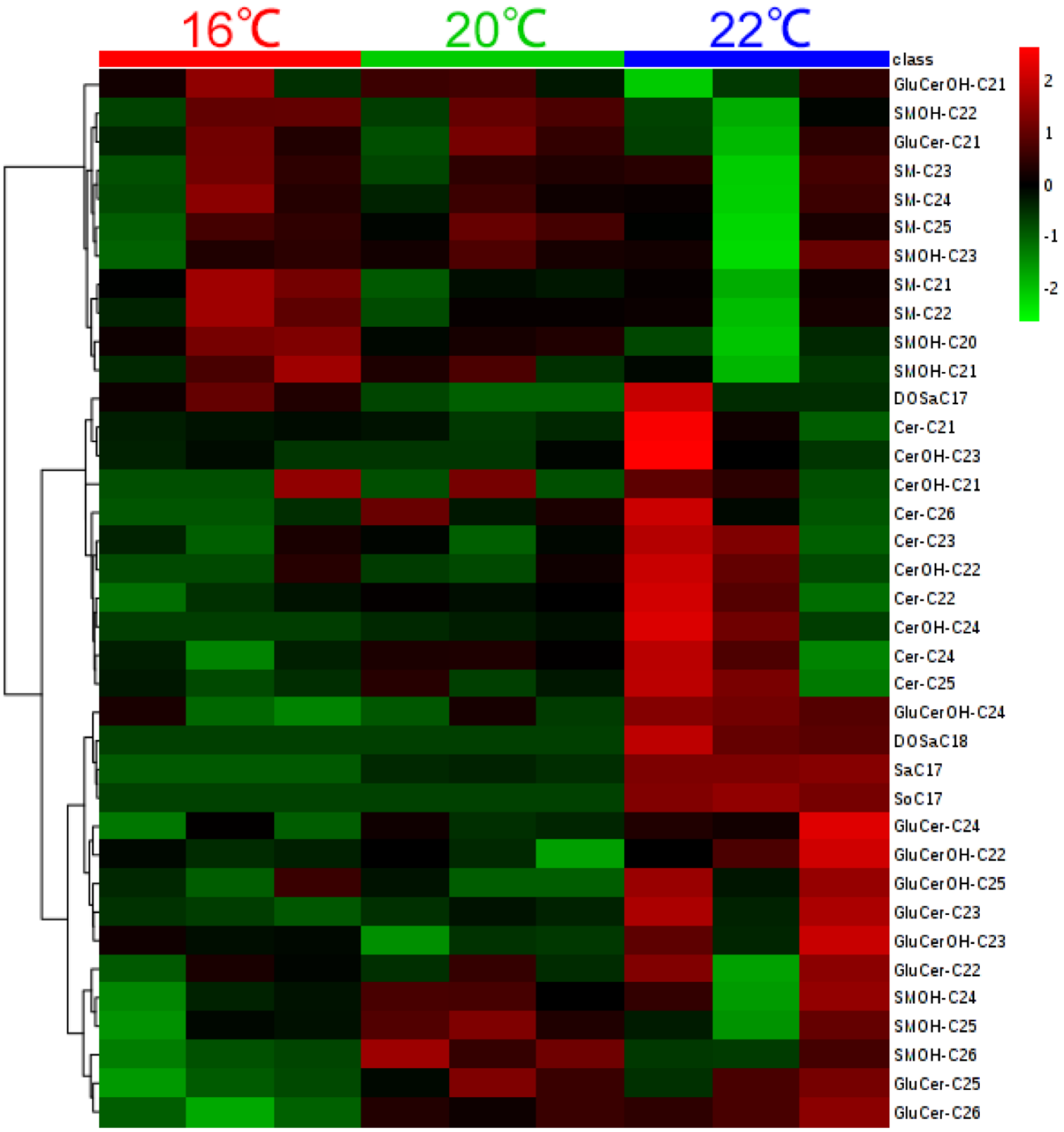

Figure S5: Hierarchical heat map showing clustering of 37 SL species quantified in N2 worms when fed with different temperature. The color of each section is proportional to the significance of alteration of compounds. Green indicates lower and red indicates higher concentrations. Columns represent individual experiments and rows represent each quantified compound.

Figure S6

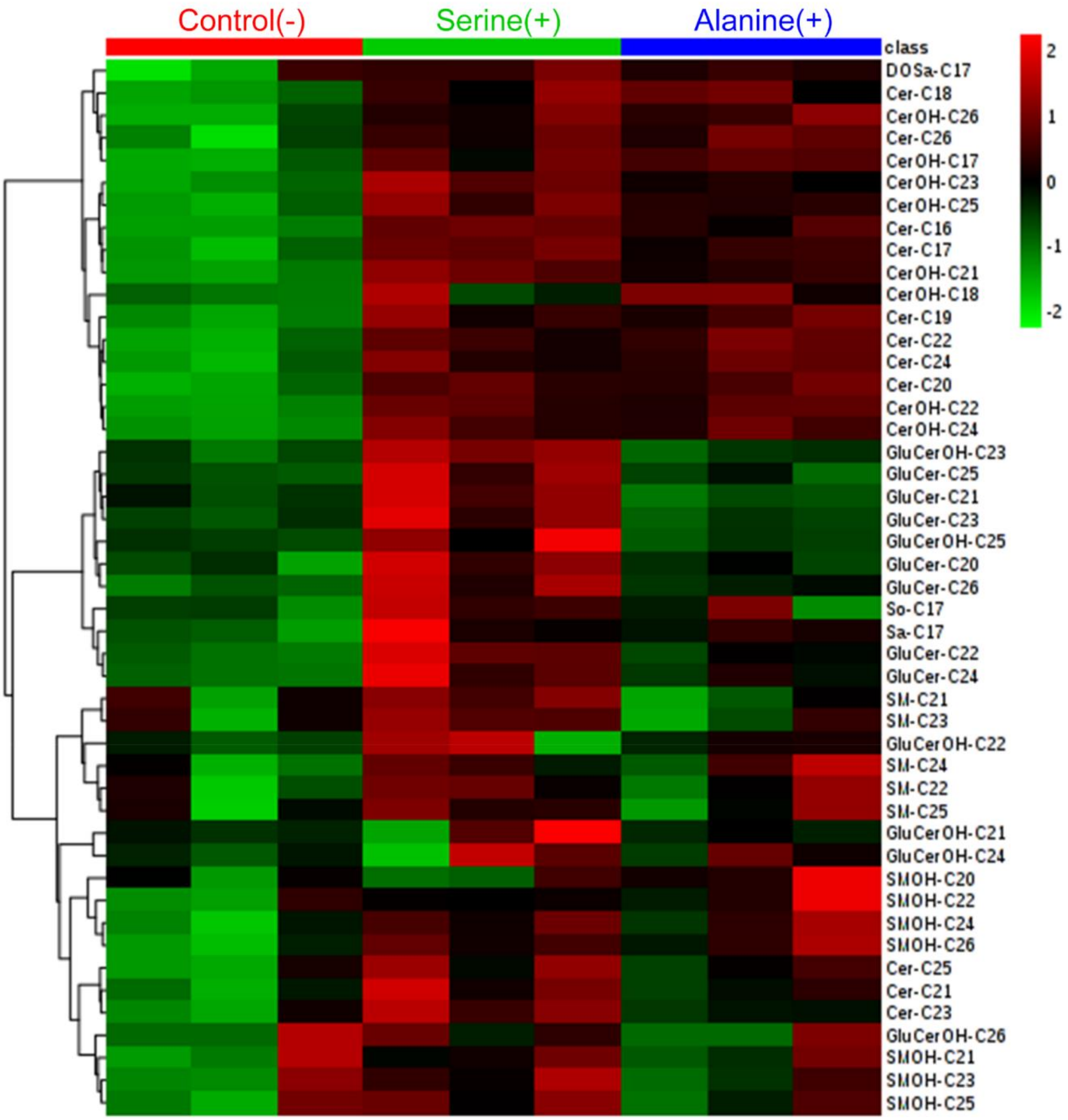

Figure S6: Hierarchical heat map showing clustering of 47 SL species quantified in N2 worms when fed with different amino acids. The color of each section is proportional to the significance of alteration of compounds. Green indicates lower and red indicates higher concentrations. Columns represent individual experiments and rows represent each quantified compound.

Table S1: Sphingolipidomic profiling of C. elegans L4-stage N2 animals.

Sample Name	Analyte Peak Name	Analyte Peak Area (counts)	Analyte Mass Ranges (amu)
ISTD[1]	Sa (d17:0)	1.48E+04	288.44/270.2
N2	Sa (d15:0)	ND	260.4360/242.4207
N2	Sa (d16:0)	ND	274.4625/256.4473
N2	Sa (d17:0)	4.99E+03	288.44/270.2
N2	Sa (d18:0)	ND	302.52/284.5
N2	Sa (d19:0)	5.34E+02	316.54/298.5
N2	Sa (d20:0)	3.27E+02	330.569/312.554
N2	Sa (d21:0)	ND	344.5954/326.5802
N2	Sa (d22:0)	ND	358.6220/340.6067
N2	Sa (d23:0)	ND	372.6486/354.6333
ISTD	So (d17:1)	6.06E+03	286.27/268.2
N2	So (d15:1)	ND	285.243/240.233
N2	So (d16:1)	ND	272.259/254.248
N2	So (d17:1)	2.78E+02	286.27/268.2
N2	So (d18:1)	ND	300.29/282.28
N2	So (d19:1)	ND	314.306/296.295
N2	So (d20:1)	ND	328.322/310.311

N2	So (d21:1)	ND	342.337/324.327
N2	So (d22:1)	ND	356.353/336.342
N2	So (d23:1)	ND	370.360/352.358
ISTD	DoSa (m18:0)	1.15E+05	286.31/268.3
N2	DoSa (m15:0)	ND	244.487/226.421
N2	DoSa (m16:0)	ND	258.463/240.448
N2	DoSa (m17:0)	1.29E+03	272.49/254.474
N2	DoSa (m18:0)	ND	286.516/268.501
N2	DoSa (m19:0)	6.36E+02	300.543/282.528
N2	DoSa (m20:0)	ND	314.57/296.554
N2	DoSa (m21:0)	ND	328.596/310.581
N2	DoSa (m22:0)	ND	342.623/324.607
N2	DoSa (m23:0)	ND	356.649/338.634
ISTD	DoMSa (m18:0)	1.47E+05	272.49/254.474
N2	DoMSa (m17:0)	ND	258.26/240.269
N2	DoMSa (m19:0)	ND	286.311/268.3
ISTD	Sa1P (d17:0)	3.18E+03	368.41/270.29
N2	Sa1P (d17:0)	2.49E+02	368.41/ 270.29
ISTD	So1P (d17:1)	6.98E+03	366.34/250.2
N2	So1P (d17:1)	7.30E+01	366.34/250.2

ISTD	Cer (d18:1/12:0)	2.79E+03	482.59/264.2
N2	Cer (d17:1/16:0)	7.80E+01	524.882/250.254
N2	Cer (d17:1/17:0)	4.03E+02	538.9086/250.254
N2	Cer (d17:1/18:0)	2.10E+03	552.9352/250.254
N2	Cer (d17:1/19:0)	5.20E+01	566.9618/250.254
N2	Cer (d17:1/20:0)	3.60E+01	580.9884/250.254
N2	Cer (d17:1/21:0)	1.68E+03	595.0149/250.254
N2	Cer (d17:1/22:0)	7.91E+03	609.0415/250.254
N2	Cer (d17:1/23:0)	2.96E+03	623.0681/250.254
N2	Cer (d17:1/24:0)	6.87E+03	637.0947/250.254
N2	Cer (d17:1/25:0)	1.76E+03	651.1213/250.254
N2	Cer (d17:1/26:0)	4.78E+02	665.1478/250.254
N2	Cer (d17:1/27:0)	1.07E+02	679.1744/250.254
N2	CerOH (d17:1/16:0)	1.24E+02	540.8814/250.254
N2	CerOH (d17:1/17:0)	2.69E+03	554.908/250.254
N2	CerOH (d17:1/18:0)	5.00E+01	568.9346/250.254
N2	CerOH (d17:1/19:0)	ND	582.9612/250.254
N2	CerOH (d17:1/20:0)	ND	596.9878/250.254
N2	CerOH (d17:1/21:0)	3.49E+02	611.0143/250.254
N2	CerOH (d17:1/22:0)	1.06E+03	625.0409/250.254

N2	CerOH (d17:1/23:0)	5.65E+02	639.0675/250.254
N2	CerOH (d17:1/24:0)	8.42E+02	653.0941/250.254
N2	CerOH (d17:1/25:0)	2.68E+02	667.1207/250.254
N2	CerOH (d17:1/26:0)	1.37E+02	681.1472/250.254
N2	CerOH (d17:1/27:0)	ND	695.1738/250.254
N2	DHCer (d17:0/16:0)	ND	526.8979/252.269
N2	DHCer (d17:0/17:0)	ND	540.9245/252.269
N2	DHCer (d17:0/18:0)	ND	554.9511/252.269
N2	DHCer (d17:0/19:0)	ND	568.9777/252.269
N2	DHCer (d17:0/20:0)	ND	583.0042/252.269
N2	DHCer (d17:0/21:0)	ND	597.0308/252.269
N2	DHCer (d17:0/22:0)	ND	611.0574/252.269
N2	DHCer (d17:0/23:0)	ND	625.084/252.269
N2	DHCer (d17:0/24:0)	ND	639.1106/252.269
N2	DHCer (d17:0/25:0)	ND	653.1371/252.269
N2	DHCer (d17:0/26:0)	ND	667.1637/252.269
N2	DHCer (d17:0/27:0)	ND	681.1903/252.269
N2	DHCerOH (d17:0/16:0)	ND	542.8973/252.269
N2	DHCerOH (d17:0/17:0)	ND	556.9239/252.269
N2	DHCerOH (d17:0/18:0)	ND	570.9505/252.269

N2	DHCerOH (d17:0/19:0)	ND	584.9771/252.269
N2	DHCerOH (d17:0/20:0)	ND	599.0036/252.269
N2	DHCerOH (d17:0/21:0)	ND	613.0302/252.269
N2	DHCerOH (d17:0/22:0)	ND	627.0568/252.269
N2	DHCerOH (d17:0/23:0)	ND	641.0834/252.269
N2	DHCerOH (d17:0/24:0)	ND	655.11/252.269
N2	DHCerOH (d17:0/25:0)	ND	669.1365/252.269
N2	DHCerOH (d17:0/26:0)	ND	683.1631/252.269
N2	DHCerOH (d17:0/27:0)	ND	697.1897/252.269
ISTD	GluCer (d17:1/12:0)	3.47E+05	644.68/264.50
N2	GluCer (d17:1/16:0)	ND	686.5571/250.3
N2	GluCer (d17:1/17:0)	ND	700.5727/250.3
N2	GluCer (d17:1/18:0)	ND	714.5884/250.3
N2	GluCer (d17:1/19:0)	ND	728.604/250.3
N2	GluCer (d17:1/20:0)	2.50E+02	742.6197/250.3
N2	GluCer (d17:1/21:0)	3.93E+03	756.6353/250.3
N2	GluCer (d17:1/22:0)	1.71E+04	770.6510/250.3
N2	GluCer (d17:1/23:0)	5.73E+03	784.6666/250.3
N2	GluCer (d17:1/24:0)	1.25E+04	798.6823/250.3
N2	GluCer (d17:1/25:0)	4.06E+03	812.6979/250.3

N2	GluCer (d17:1/26:0)	1.15E+03	826.7136/250.3
N2	GluCer (d17:1/27:0)	ND	840.7292/250.3
N2	GluCerOH (d17:1/16:0)	ND	702.552/250.3
N2	GluCerOH (d17:1/17:0)	ND	716.5677/250.3
N2	GluCerOH (d17:1/18:0)	ND	730.5833/250.3
N2	GluCerOH (d17:1/19:0)	ND	744.599/250.3
N2	GluCerOH (d17:1/20:0)	ND	758.6146/250.3
N2	GluCerOH (d17:1/21:0)	9.48E+02	772.6303/250.3
N2	GluCerOH (d17:1/22:0)	3.71E+03	786.6459/250.3
N2	GluCerOH (d17:1/23:0)	1.52E+03	800.6616/250.3
N2	GluCerOH (d17:1/24:0)	3.02E+03	814.6772/250.3
N2	GluCerOH (d17:1/25:0)	5.95E+02	828.6929/250.3
N2	GluCerOH (d17:1/26:0)	1.38E+03	842.7085/250.3
N2	GluCerOH (d17:1/27:0)	ND	856.7242/250.3
ISTD	SM (d18:1/12:0)	1.11E+06	647.9/184.2
N2	SM(d17:1/16:0)	1.44E+04	690.5676/184.2
N2	SM(d17:1/17:0)	3.15E+04	704.5832/184.2
N2	SM(d17:1/18:0)	1.30E+04	718.5989/184.2
N2	SM(d17:1/19:0)	1.44E+04	732.6145/184.2
N2	SM(d17:1/20:0)	2.52E+04	746.6302/184.2

N2	SM(d17:1/21:0)	1.10E+05	760.6458/184.2
N2	SM(d17:1/22:0)	5.94E+05	774.6615/184.2
N2	SM(d17:1/23:0)	1.71E+05	788.6771/184.2
N2	SM(d17:1/24:0)	1.04E+05	802.6928/184.2
N2	SM(d17:1/25:0)	3.67E+04	816.7084/184.2
N2	SM(d17:1/26:0)	2.04E+04	830.7241/184.2
N2	SM(d17:1/27:0)	2.58E+03	844.7397/184.2
N2	SMOH(d17:1/16:0)	ND	706.5625/184.2
N2	SMOH(d17:1/17:0)	ND	720.5781/184.2
N2	SMOH(d17:1/18:0)	ND	734.5938/184.2
N2	SMOH(d17:1/19:0)	ND	748.6094/184.2
N2	SMOH(d17:1/20:0)	1.67E+04	762.6251/184.2
N2	SMOH(d17:1/21:0)	2.53E+05	776.6407/184.2
N2	SMOH(d17:1/22:0)	1.22E+06	790.6564/184.2
N2	SMOH(d17:1/23:0)	3.53E+05	804.6720/184.2
N2	SMOH(d17:1/24:0)	5.15E+05	818.6877/184.2
N2	SMOH(d17:1/25:0)	1.63E+05	832.7033/184.2
N2	SMOH(d17:1/26:0)	8.49E+04	846.7190/184.2
N2	SMOH(d17:1/27:0)	ND	860.7346/184.2
N2	DHSM(d17:0/16:0)	ND	692.583/252.269

N2	DHSM(d17:0/17:0)	ND	706.599/252.269
N2	DHSM(d17:0/18:0)	ND	720.615/252.269
N2	DHSM(d17:0/19:0)	ND	734.630/252.269
N2	DHSM(d17:0/20:0)	ND	748.646/252.269
N2	DHSM(d17:0/21:0)	ND	762.662/252.269
N2	DHSM(d17:0/22:0)	ND	776.677/252.269
N2	DHSM(d17:0/23:0)	1.90E+02	790.693/252.269

[1] ISTD: Internal standard (see Methods)
